# Photosensitizer spatial heterogeneity and its impact on personalized interstitial photodynamic therapy treatment planning

**DOI:** 10.1117/1.JBO.30.1.018001

**Published:** 2025-01-11

**Authors:** Tina Saeidi, Shuran Wang, Hector A. Contreras, Michael J. Daly, Vaughn Betz, Lothar Lilge

**Affiliations:** a University of Toronto, University Health Network, Princess Margaret Cancer Centre, Department of Medical Biophysics, Toronto, Ontario, Canada; b University of Toronto, Edward S. Rogers Sr. Department of Electrical and Computer Engineering, Toronto, Ontario, Canada; c University Health Network, Princess Margaret Cancer Centre, Toronto, Ontario, Canada

**Keywords:** interstitial photodynamic therapy, pre-treatment planning, PDT-SPACE, photosensitizer heterogeneity

## Abstract

**Significance:**

Personalized photodynamic therapy (PDT) treatment planning requires knowledge of the spatial and temporal co-localization of photons, photosensitizers (PSs), and oxygen. The inter- and intra-subject variability in the photosensitizer concentration can lead to suboptimal outcomes using standard treatment plans.

**Aim:**

We aim to quantify the PS spatial variation in tumors and its effect on PDT treatment planning solutions.

**Approach:**

The spatial variability of two PSs is imaged at various spatial resolutions for an orthotopic rat glioma model and applied *in silico* to human glioblastoma models to determine the spatial PDT dose, including in organs at risk. An open-source interstitial photodynamic therapy (iPDT) planning tool is applied to these models, deriving the spatial photosensitizer quantification resolution that consistently impacts iPDT source placement and power allocation.

**Results:**

The *ex vivo* studies revealed a bimodal photosensitizer distribution in the tumor. The concentration of the PS can vary by a factor of 2 between the tumor core and rim, with slight variation within the core but a factor of 5 in the rim. An average sampling volume of $1\;{\mathrm{mm}}^{3}$ for photosensitizer quantification will result in significantly different iPDT planning solutions for each case.

**Conclusions:**

Assuming homogeneous photosensitizer distribution results in suboptimal therapeutic outcomes, we highlight the need to predict the photosensitizer distribution before source placement for effective treatment plans.

## 1 Introduction

Photodynamic therapy (PDT) is a minimally invasive treatment modality used for various indications in oncology,^1^ infectious condition,^2^ and cosmetic applications.^3^ PDT uses a light-activated drug called photosensitizer (PS) that preferentially accumulates in highly proliferating tissues, such as malignancies. Upon activation by light, the PS generates reactive oxygen species (ROS), including singlet oxygen, which leads to localized tissue destruction through mechanisms such as necrosis, apoptosis, vascular damage, and immune response activation.^4^^–^^7^

The local tissue response is given when the local cytotoxic ROS concentration exceeds the scavenging and protein replacement ability of cells. The ROS concentration is given by the spatial and temporal overlap of the PS concentration (PS), its extinction coefficient (ε), at the treatment wavelength (λ), and ROS quantum yield, the photon density (hν), and a minimum molecular oxygen concentration [O32]. The product is a commonly used PDT dose definition.^8^ The PS accumulation and oxygen concentration in the clinical target volume (CTV) and the surrounding organs at risk (OARs) can vary between and within patients. Heterogeneity in PS accumulation, primarily attributed to irregular vascular patterns, often results in variability in treatment response and limits treatment efficacy, particularly in interstitial PDT (iPDT).^9^^–^^12^ Other factors, such as tumor oxygenation and percentage of hypoxia, impact the treatment efficacy; however, the [PS] and [O32] are entered as a multiplicative effect for the PDT efficacy dose.^11^ Thus, the photon density or fluence rate may need to be adjusted locally based on all efficacy determining factors for each patient to avoid under- or over-dosing of the CTV or the adjacent OAR, respectively.

In superficial applications, where small tumors or pre-malignant conditions are targeted, no significant variations in [PS] and [O32] are observed throughout the CTV, given the often topical PS delivery.^13^^,^^14^ An empirical treatment plan relying solely on the PS and its administered concentration, the optical power density ($\mathrm{mW}\cdot {\mathrm{cm}}^{-2}$), and irradiation time can achieve satisfactory PDT outcomes for superficial organs.^15^^–^^17^ For iPDT, current treatment protocols rely on empirical guidelines that specify fixed distances between cylindrical diffusing tip fibers within the CTV based on a homogeneous PS accumulation assumption. Under this assumption, iPDT planning guidelines address complex shapes and sizes of the CTV to achieve the target PDT dose while preventing over-treatment of surrounding host tissue and OAR.^18^^,^^19^ However, applying this homogenous model to clinical scenarios with heterogenous PS accumulation may result in undesirable treatment outcomes and potentially harm vital organs.

As reported over the past two decades, in preclinical studies for some superficial applications^20^^,^^21^ and more solid tumors,^22^^,^^23^ a significant fraction of non-responders to PDT presented with a lack of PS accumulation, particularly for protoporphyrin IX (PPIX)-mediated PDT when the tumor fails to synthesize it from the precursor or otherwise shows significant variability in local [PS] within the CTV.^24^ Johansson et al.^24^ found a strong correlation between PPIX fluorescence in brain tumors and long-term survival and suggested in subsequent research that individualizing PDT parameters, such as PPIX fluorescence and photobleaching, could improve clinical outcomes.^25^ They also noted that small, well-vascularized tumors responded better to iPDT.^26^ Other studies emphasized the importance of local PS and oxygen availability for treatment efficacy, with recommendations to quantify PS levels and adjust light doses to reduce variability and enhance consistency in clinical results.^11^^,^^27^ Currently, quantifying [PS] as a function of position, r, can be achieved by online monitoring of the PS’s fluorescence emission. Treatment monitoring during iPDT is based on qualitative or quantitative fiber optic-based^28^ and/or widefield PS fluorescence intensity measurements.^29^^,^^30^ However, these approaches come with their own strengths and limitations, depending on the application. Most PS dosimetry systems integrate over large tissue volumes and focus on [PS] differences between the subjects. These systems tend to undersample the CTV, limiting their utility for treatment planning.^11^ Many methods are restricted to superficial regions at the exterior of the CTV, assuming that the surface measurements represent the target’s overall heterogeneity, which may not accurately reflect the [PS] distribution within larger tumors that contain hypoxic, anoxic, or necrotic tissue.^11^ Higher resolution techniques, such as those utilizing multiple optical fibers like the approach used in SpectraCure, may work well in homogeneous tissues like the prostate^31^^,^^32^ due to the structure of the vascular perfusion tree, which confines the light within the prostate gland and minimizes the damage to OAR. However, these techniques are less effective in heterogeneous tissues with varying vascular development, such as in the brain, leading to poor treatment quality and under-treatment of the CTV.^24^ In addition, in some cases, such as pancreatic cancer, fiber-based fluorescence assays can be challenging or infeasible to place, resulting in incomplete [PS] information across the CTV.^12^

Various PDT dose models have been proposed for treatment planning, maximizing the CTV receiving a sufficiently high PDT dose to cause the desired biological effect while minimizing the volume of surrounding OARs.^33^^–^^35^ Among these, PDT-SPACE^36^^–^^40^ employs the photodynamic threshold model,^33^ which includes the specific tissue responsivity for a given PS, distinguishing it from other approaches, such as ROS concentration-based models^34^^,^^35^ or fluence and fluence-rate-based models^41^^,^^42^ as used in alternative systems. Regardless of the model used, the treatment plan aims to distribute the photon sources throughout the CTV, accounting for co-variables such as [O32] and photobleaching rates, to achieve the destruction of the CTV while preserving the surrounding healthy tissues, particularly the OAR.

In prior work, Yassin et al.^43^ considered the variation in the optical properties in the optimization protocols (PDT-SPACE) for interstitial photon source placement. This consideration was incorporated when calculating the forward solution based on Monte Carlo simulations (FullMonte).^36^^–^^39^^,^^44^^,^^45^ However, the PDT-SPACE program does not account for local variations in [PS], and hence, local absorption coefficient changes. It is currently unknown how [PS] heterogeneity would impact the treatment plan and therapeutic outcome if considered during the planning process. In addition, the minimal spatial resolution, Δvolume, over which the $\left[\overset{¯}{PS}\right]$ varies significantly and can impact PDT planning optimization, and hence, treatment efficacy is uncertain.

Here, we investigate the spatial variability of the [PS] distribution for two photosensitizers (PSs) (Ce6 and Porphysome) in rat brain RG2 (rat glioma) tumor models. Human brain tumor simulation models were generated at various spatial resolutions to examine the influence of assumed heterogeneous versus homogeneous [PS] distributions on iPDT pre-treatment planning program (PDT-SPACE) outcome, including source placement and optical power delivery. Insights from the rat models were applied to the human simulations to explore the impact of heterogeneity in larger, more complex, human-sized tumors. We determined the spatial resolution necessary for determining Δ[PS], which can lead to clinically significant modifications in treatment plans and potential improvement in treatment outcomes.

## 2 Methods

### 2.1 Drugs

This study used a molecular-based PS, chlorin e6 (Fotolon, Synverdis, Germany) [Ce6], and a nanoparticle-based chlorin e6 analog, named, Porphysome. Talaporfin, a molecular chlorin e6 product, has been approved for lung cancer and glioma in Japan.^46^^,^^47^ The two versions of chlorin e6 employed here presented varying extravasation, diffusion coefficients, and intratumoral accumulation to illustrate the potential variation in the spatial resolution of [PS] variations for different PSs.

Porphysomes were provided courtesy of Dr. Zheng’s Princess Margaret Cancer Centre group. chlorin e6 has a molecular weight of $596.6\;g\cdot {\mathrm{mol}}^{-1}$. Porphysomes are self-assembled lipid nanoparticles (30 to 120 nm) made of pyropheophorbide-conjugated phospholipids holding ∼80,000 chlorin PSs. The molecular weight of the pyro-lipid is $1012.3\;g\cdot {\mathrm{mol}}^{-1}$, and the molecular weight of only the pyro (pyropheophorbide-a) is $534.7\;g\cdot {\mathrm{mol}}^{-1}$. They are internalized into cells via folate–receptor-mediated endocytosis, disrupting their nanostructure, creating chlorin monomers for PDT,^48^^,^^49^ and other imaging or therapy-related applications.^50^ Nanoparticle PSs can offer enhanced permeability and retention in the tumor compared with molecular-based PSs.^51^

### 2.2 *In Vivo* Study

#### 2.2.1 Animal and tumor model

All animal procedures were approved by the University Health Network’s (UHN) Institutional Animal Care Committee (ARC) and adhered to the guidelines of the Canadian Council for Animal Care (Animal Use Protocol #6514). RG2 tumors were grown intracranially in the brains of F344/NHsd rats as models to image the spatially resolved PS accumulation in the tumors and contralateral normal brain tissue. Eight rats were injected intracranially with 5000 cells suspended in 5  μL via a burr hole; see Refs. 52 and 53 for surgical details. After a growth period of 20 to 22 days, four animals received $10\;\mathrm{mg}\cdot {\mathrm{kg}}^{-1}$ of porphysomes equivalent to $∼5\;\mathrm{mg}\cdot {\mathrm{kg}}^{-1}$ of the PS monomer (group A), and four animals were i.v. injected with $3\;\mathrm{mg}\cdot {\mathrm{kg}}^{-1}$ of Ce6 (group B). Rats administered Ce6 were euthanized 3 h post-injection as this time interval has previously been shown to have the highest specific uptake ratio between malignancies and normal host tissue, also representing the clinically standard drug light interval. Rats administered with porphysome were euthanized 24 h post-injection. The animal brains were extracted, snap-frozen in liquid nitrogen-cooled isopropanol, cut into 1- or 2-mm tissue slices, and kept on ice to prevent drying before imaging. Following PS fluorescence imaging, samples were fixed in 10% buffered formalin for standard histological analysis. Twenty-one brain slices containing tumors were collected for group A, and 19 slices were collected for group B.

#### 2.2.2 Histopathology

Histopathological evaluation of the tumor growth was carried out on hematoxylin and eosin-stained, 4-μm sections from both sides of the brain slices for further processing at the STTARR Innovation Centre Pathology Core and scanned using a whole slide brightfield microscope [Aperio AT2 brightfield scanner (Leica Biosystems)]. Five slices (out of 40 total slices from eight rats) were excluded due to thin cuts and deformation during imaging and kept aside for macroscopic [PS] quantification based on tissue extraction.^54^

#### 2.2.3 PS quantification

Quantitative spatial frequency domain imaging (qSFDI) was used as the “gold” standard for *ex vivo* spatially resolved [PS] determination at a single time point post-injection.^55^^,^^56^ qSFDI utilizes three sinusoidal illumination structures with a 120-deg phase shift between them to calculate the intensity-modulated reflectance at each sample point, $M_{\mathrm{AC}}^{\text{sample}}$, according to Eq. (1)^56^

$$M_{\mathrm{AC}}^{\text{sample}}(r)=\frac{\sqrt[{2}]{2}}{3}\sqrt[{2}]{\left((I_{1}(r)-I_{2}(r){)}^{2}+(I_{2}(r)-I_{3}(r){)}^{2}+(I_{3}(r)-I_{1}(r){)}^{2}\right)}.$$ (1)

The benchtop SFDI system used in this study was comprised of a 14-bit monochrome charge-coupled device camera, a 1080p digital light projector (DLP), a light engine with six light-emitting diodes, a 25-mm focal-length lens, and crossed linear polarizers.^57^^,^^58^ Using 438-nm excitation and 668-nm fluorescence emission wavelengths, the tissues’ absorption ($\mu_{a}$) and reduced scattering ($\mu_{s}^{\prime }$) coefficients and the measured PS fluorescence intensity, F, [PS] were determined as a function of distance, r, based on the measured local reflectance, R, at the excitation, $R_{x}$, and emission, $R_{m}$, wavelengths, and the emitted fluorescence intensity, $F_{m}$, relative to known reference samples, $M_{AC}^{\mathrm{ref}}$, according to light diffusion theory; see Eq. (2)^56^^,^^59^

$$[\mathrm{PS}](r)=k(\mathrm{PS})\frac{\mu_{a,x}F_{m}(r)}{1-(1-\frac{M_{\mathrm{AC}}^{\text{sample}}(r)}{M_{\mathrm{AC}}^{\mathrm{ref}}(r)}R_{x}^{\mathrm{ref}}(r))\frac{M_{\mathrm{AC}}^{\text{sample}}(r)}{M_{\mathrm{AC}}^{\mathrm{ref}}(r)}R_{m}^{\mathrm{ref}}(r)},$$ (2)

where k(PS) scales to concentration units (${\mu g\cdot \mathrm{mL}}^{-1}$) using the PS-specific fluorescence absorption coefficient and quantum efficiency. Three spatial imaging frequencies (f) 0, 0.2, and $0.5\;{\mathrm{mm}}^{-1}$ were employed, whereby $0\;{\mathrm{mm}}^{-1}$ quantifies [PS] equivalent to standard epi-fluorescence macroscopy, whereas higher spatial frequencies result in shallower depth sensitivity. From the Monte Carlo analysis of Hayakawa et al.^60^ and assuming a nominal albedo of $\mu_{s}/\mu_{a}=1$ ($\mu_{s}$ is the scattering coefficient), the sampling depths vary from $1.2\;{\mathrm{mm}}^{-1}$ for f=0 to 0.5 mm for $f=0.5\;{\mathrm{mm}}^{-1}$.^56^^,^^60^ However, this depth is influenced by the laser wavelength; in this case, 438 nm results in a penetration depth of less than 1 mm.^61^ Therefore, for all subsequent analyses, only the spatial frequency of $0.5\;{\mathrm{mm}}^{-1}$ was selected due to its highest spatial resolution and considering the limited penetration depth of the 438-nm excitation wavelength.

Each tissue slice was imaged from both sides, denoted as “side 1” and “side 2”, respectively, with a pixel resolution of 0.06×0.06  mm. The qSFDI system’s sensitivity was established using serial dilutions of the fluorophore Ce6 in liquid phantoms comprised of India ink and Intralipid ($\mu_{a}=0.2\;{\mathrm{mm}}^{-1}$, $\mu_{s}^{\prime }=2\;{\mathrm{mm}}^{-1}$), yielding a minimum detection threshold of 0.01  μg/mL Ce6. The average [PS] in regions of interest was validated through tissue solubilization and fluorescence quantification.^54^

#### 2.2.4 Texture analysis

Texture analysis^62^ was used to gain insights into the spatial distribution of a PS within an image, providing metrics to describe intralesional heterogeneity. Various statistical, model-based, and transform-based approaches are available.^63^^,^^64^ Here, histogram analysis and the gray level co-occurrence matrix (GLCM), a widely used tool in texture analysis, are employed in statistical-based techniques. Histogram analysis provides insights into the frequency distribution of pixel intensities within a region of interest. GLCM, derived from co-occurrence measurements, captures the relationships among pixel intensities, offering a comprehensive view of the texture properties of the image.

#### 2.2.5 Histogram analysis

qSFDI images were acquired for the brain slices of groups A and B animals; MATLAB codes generate histograms and density plots, illustrating the range of heterogeneity within and between qSFDI photosensitizer concentrations [PS] in the rat brain tissue for the two regions of interest (ROIs) representing tumor and contralateral normal tissue, respectively. Multiple slices for the four animals in each group provided data to analyze the intra- and inter-animal variation in each group, followed by comparison between groups. Otsu’s thresholding method, implemented in MATLAB using the otsuthresh(counts) function, was used to correlate different modes of the density plot with spatial variation in [PS] within the tumor. This technique analyzes the image histogram to determine the optimal threshold that separates the foreground from the background by maximizing between-class variance and minimizing within-class variance for effective segmentation.

#### 2.2.6 GLCM analysis

The acquired qSFDI images were also analyzed using a GLCM texture analysis approach at different spatial resolutions to give further insight into the distribution of [PS] values and its heterogeneity in the tumor location. This approach was implemented here to study the sensitivity of the PDT-SPACE optimization tool to different scales of [PS] spatial heterogeneity. The simulation models are then generated at these different resolutions accordingly. An in-house code written in MATLAB was used for ROI selection within the image, followed by GLCM and textural features calculations as proposed by Haralick et al.^65^ Among the available set of Haralick features, three parameters of interest were measured: contrast, energy, and homogeneity, as these are expected to be sensitive to the [PS] distribution variability in both healthy and tumor tissue. The number of levels for GLCM calculations was kept constant across all the images, and the [PS] dynamic range was defined according to the histograms of the qSFDI images. The neighboring pixels for GLCM calculations are defined by angle directions (e.g., 0, 45, 90, and 135 deg). These measurements were repeated by changing the window size (1, 5, 15, and 20 pixel window size). The final output of the approach is a list containing the features of interest for every pixel in the ROI. Box and whisker plots are then generated as a function of window size. The central pixel of the sliding window (step size = 1) is considered the pixel of interest for the calculations.^66^

**Fig. 1:**
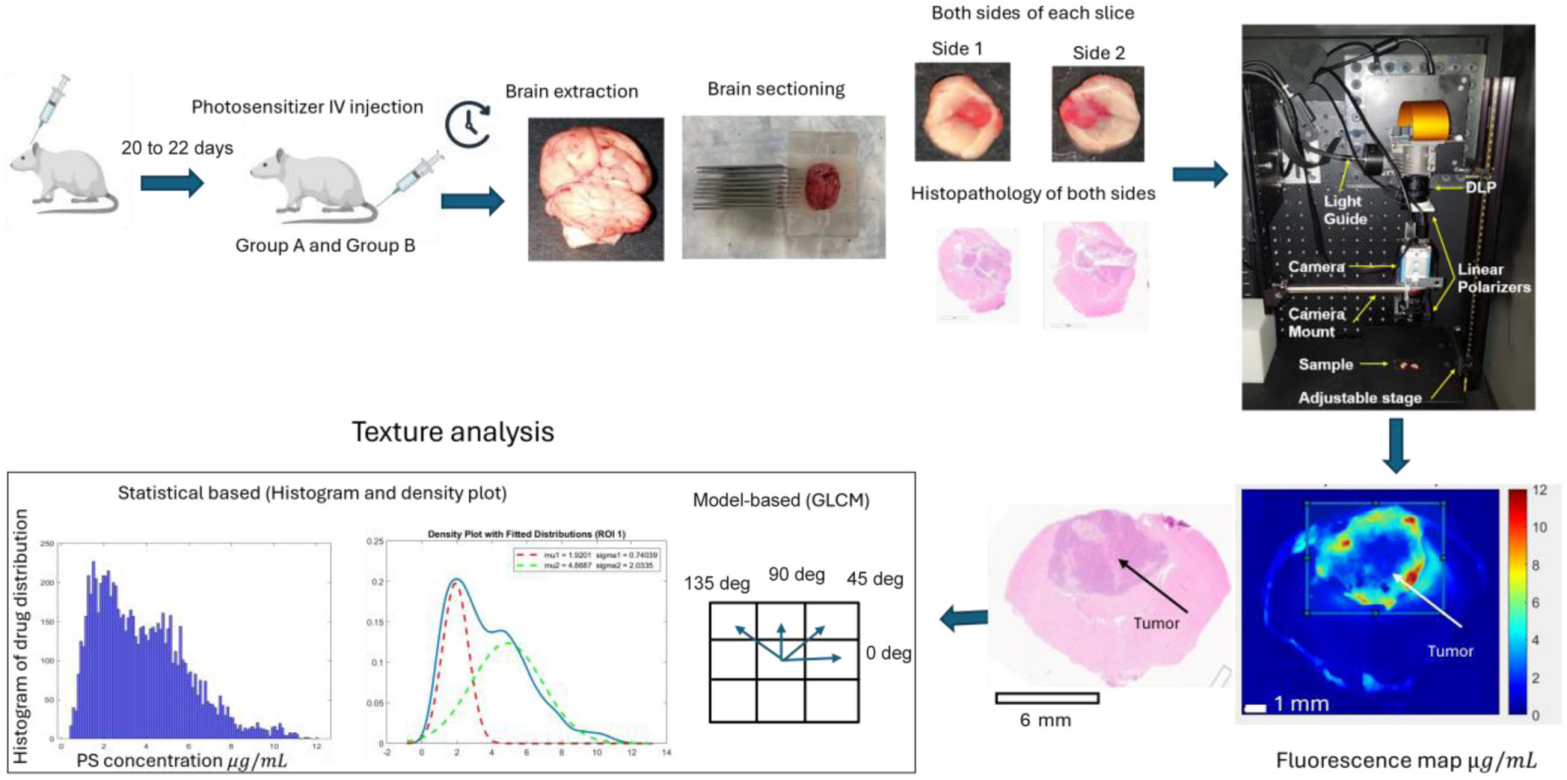
Experimental workflow showing the steps of our methodology, including intracranial tumor induction and intravenous injection of the animals (group A received $10\;\mathrm{mg}\;{\mathrm{kg}}^{-1}$ porphysome, and group B received 3  mg/kg Ce6). Group A animals were euthanized 24 h after injection, and group B animals were euthanized 3 h after injection. Brains were extracted, sectioned, and imaged on both sides of each slice by qSFDI. Histopathology was conducted to identify tumors. Fluorescence imaging was performed to quantify [PS] within the tumor and normal tissues, followed by histogram and density plot analyses of drug concentrations in both regions of interest (ROIs). Texture analysis was performed for both statistical and model-based evaluations.

Figure 1 summarizes a detailed overview of the animal preparation process, data collection procedures, and subsequent analysis steps described in this section.

### 2.3 Simulation Study (Origin of the Models)

To bridge the gap between preclinical animal studies and clinical applications, we generated human brain tumor models inspired by the heterogeneity observed in animal models. This approach allows us to investigate the effects of heterogeneity on iPDT treatment planning in human-sized tumors, which exhibit much larger physical volumes. Using rat models, we gain valuable insights into the scale and patterns of heterogeneity, which are crucial for developing accurate and effective treatment plans for human tumors.

*In silico* brain tumor models of various spatial resolutions were generated to explore the required resolution in determining [PS](r) to affect the treatment planning software (PDT-SPACE)^36^^,^^45^ solutions by generating distinct plans for most cases when executed as homogeneous versus heterogeneous PS distributions. These simulations use additional tumor models inspired by the texture analysis for *in vivo* models.

Two sets of test 3D tetrahedral meshes are used, including three low-resolution and three high-resolution models. The low-resolution mesh models of a human brain are taken from Refs. 67 and 68. Tumors generated in ovoid or aggregated spherical shapes to approximate clinical glioblastoma multiforme appearances in the cancer imaging archive,^69^ with average and median tetrahedral volumes of 5.39, $4.29\;{\mathrm{mm}}^{3}$, respectively, were added to the brain models. High-resolution models were segmented from clinical human T1-weighted brain MRI images (256×256×160  voxels). An open-source segmentation software based on a maximum-a-posteriori classifier algorithm developed by Shattuck et al.^70^^,^^71^ was employed to classify the regions voxel-by-voxel. A custom-made Python code artificially creates irregular brain tumors with $0.08\;{\mathrm{mm}}^{3}$ average and $0.02\;{\mathrm{mm}}^{3}$ median tetra volume within a randomly selected volume by generating several interconnected, randomly placed spheres around a central core. The total tumor volumes are comparable to the sizes of glioma tumors diagnosed in humans (see Table S1 in the Supplementary Material). Random heterogeneities two to three times higher and lower than the average [PS] were manually added to the tumor to generate heterogeneous tumor photosensitizer concentrations. Every segmented tissue in the modified volume of interest (VOI) is exported as tetrahedral VTK files using ITK-SNAP [Penn Image Computing and Science Laboratory (PICSL) University of Pennsylvania.V.3.8.0].^72^ Additional spherical and non-spherical model tumor shapes were generated with intermediate resolutions. The average and median tetra volumes were 6.99 and $3.18\;{\mathrm{mm}}^{3}$, and 3.43 and $1.05\;{\mathrm{mm}}^{3}$, for the spherical and non-spherical models, respectively. The models are generated using the technique mentioned above, representing three scenarios with heterogeneity at various physical length scales according to the results of the *in vivo* studies. The heterogeneities were manually incorporated into the tumors using Paraview (Sandia National Labs, Kitware Inc., Clifton Park, New York, United States, and Los Alamos National Labs, Los Alamos, New Mexico, United States, V.5.6.0) and an in-house-developed Python code.

The first scenario presented equal homogeneity of [PS] in the tumor core and rim. In the second, they were presented with different but homogenous concentrations in the core and the rim, with the [PS] in the core being half of that in the rim. The third presented average [PS] in the core being half of the rim incorporating heterogeneous concentration distributions within the rim, with four different heterogeneous patterns generated. All models’ average [PS] within the entire tumor, including the core and the rim, was equal. Tissue optical properties at 635 nm are used for all *in silico* simulations.^36^^,^^73^^,^^74^

**Fig. 2:**
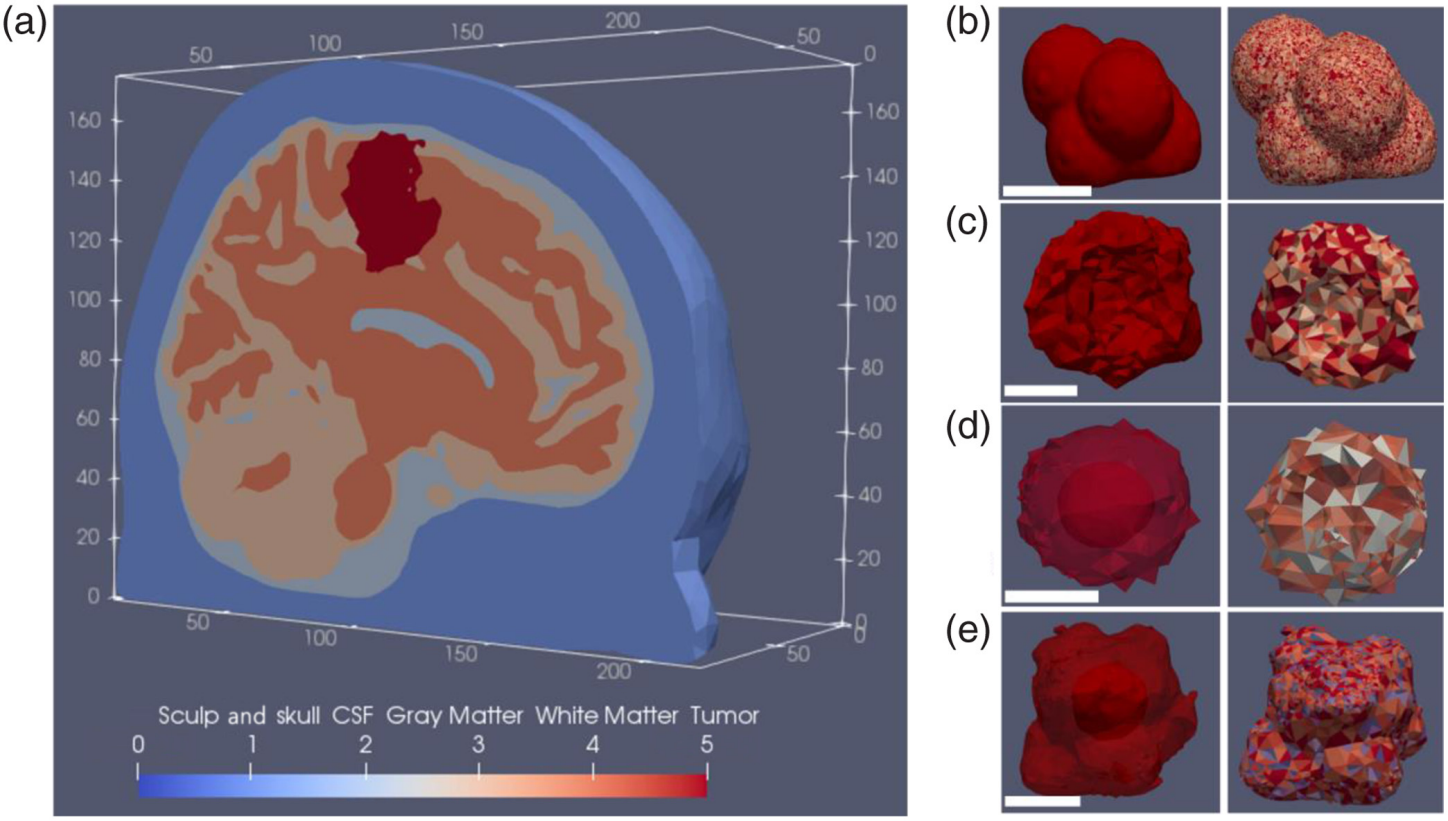
(a) Segmented human brain consisting of five regions: scalp and skull, cerebrospinal fluid (CSF), gray matter (GM), white matter (WM), and tumor. The simulation automatically adds a region 0, a bounding box surrounding the model, and terminates all the photons hitting it. (Right) shows examples of homogeneous and heterogeneous tumor models, including (b) high resolution, (c) low resolution, (d) medium resolution in a spherical shape with a necrotic core comparable to a real-case scenario, and (e) medium resolution in a non-spherical shape with a necrotic core. The solid red color is used for homogeneous [PS]. In contrast, multiple colors in the heterogeneous models show variation in [PS] by predetermined factors compared with the average [PS] in the homogeneous model. Dimensions are given in millimeters (mm), and the white scale bars correspond to 20 mm.

Figure 2 shows an example of an *in silico* model comprising five tissues—skull, cerebrospinal fluid (CSF), gray matter, white matter, and tumor, including low-, medium-, and high-resolution tumors. Figure S1 in the Supplementary Material shows all high- and low-resolution models; Fig. S2 in the Supplementary Material shows the medium-resolution models with a necrotic core comparable to real-case scenarios.

Table 1 summarizes the optical properties used for the simulation study, specifically at a wavelength of 635 nm.

**Table 1:** Summary of optical properties for 635 nm given in ${\mathrm{mm}}^{-1}$.^36^^,^^73^^,^^74^

Tissue type	$\mu_{a}$	$\mu_{s}^{\prime }$	G	n
Tumor	0.18	10	0.8	1.39
Grey matter	0.01	8.8	0.89	1.36
White matter	0.08	40.7	0.84	1.467
Scalp and skull	0.55	10.8	0.8	1.56
CSF	0.0038	0.005	0.87	1.3891

Tumor volumes and number of light sources are summarized in Table S1 in the Supplementary Material. The sources are placed within the tumor, spaced 1 cm apart from each other, and located 0.8 to 1 cm away from the tumor’s edge.

### 2.4 Computational Software

The PDT-SPACE algorithm, described previously,^36^^,^^40^ aims to exceed the PDT threshold, causing necrosis in 98% of the CTV while minimizing the OAR volume at risk of damage. The PDT-SPACE software comprises a source position optimization module using simulated annealing (SA) followed by a power optimization component based on convex optimization techniques, which allocates power to light sources based on their positions. As PDT-SPACE is a simulation-based pre-treatment planning tool, theoretical tissue destruction is assessed by comparing the simulated light dose at each tissue element to its death threshold. The PDT-SPACE power optimization algorithm iteratively adjusts based on predicted tumor destruction until it achieves exactly 98% destruction, as determined by the program. The output includes the position of each source, the power allocated to each source, its length, the resulting dose-volume histogram for the tumor, and anticipated volumetric damage in cm^3^ for the healthy tissues (V100).

For the SA simulations in PDT-SPACE, a random seed between 1 and 10 was used without explicitly accounting for variability in the results. However, Wang et al.^38^ demonstrated that the coefficient of variation (CV) is less than 0.05% for achieving the primary goal of 98% tumor destruction. For OAR damage, the CV ranged from 7.57% to 24.66% when using 10 different seeds across a variety of tumor geometries when limiting the Monte Carlo simulations to $10^{6}$ photon packages. Importantly, the magnitude of the CV was inversely proportional to the volume of the OAR. In our simulations, we used ($10^{8}$) photon packets to minimize variability further. For power optimization with fixed source positions, a single seed with the same random number generator sequence was used. This approach ensures consistent photon paths across runs, and as a result, no variations between runs are expected. PDT-SPACE simulations aim to achieve two objectives: first, to identify the spatial scale at which heterogeneity in [PS] influences the PDT-SPACE solution versus the current assumption of homogeneous [PS], and second, to analyze the variations in the PDT-SPACE solutions for the different PS distributions observed in the *in vivo* imaging studies.

For the first objective, PDT-SPACE was executed for three low- and three high-resolution tumor models, each assuming homogeneous or heterogeneous [PS] cases, optimizing the source power and position. For the second objective, PDT-SPACE was executed for all three scenarios with heterogeneity at various physical length scales outlined in the previous section. Subsequently, the power allocation solution and the differential damage to the OAR were compared.

## 3 Results

### 3.1 Characterization of Photosensitizer Heterogeneity in RG2 Tumor Within Rat Brain

#### 3.1.1 Histogram and density plots

**Fig. 3:**
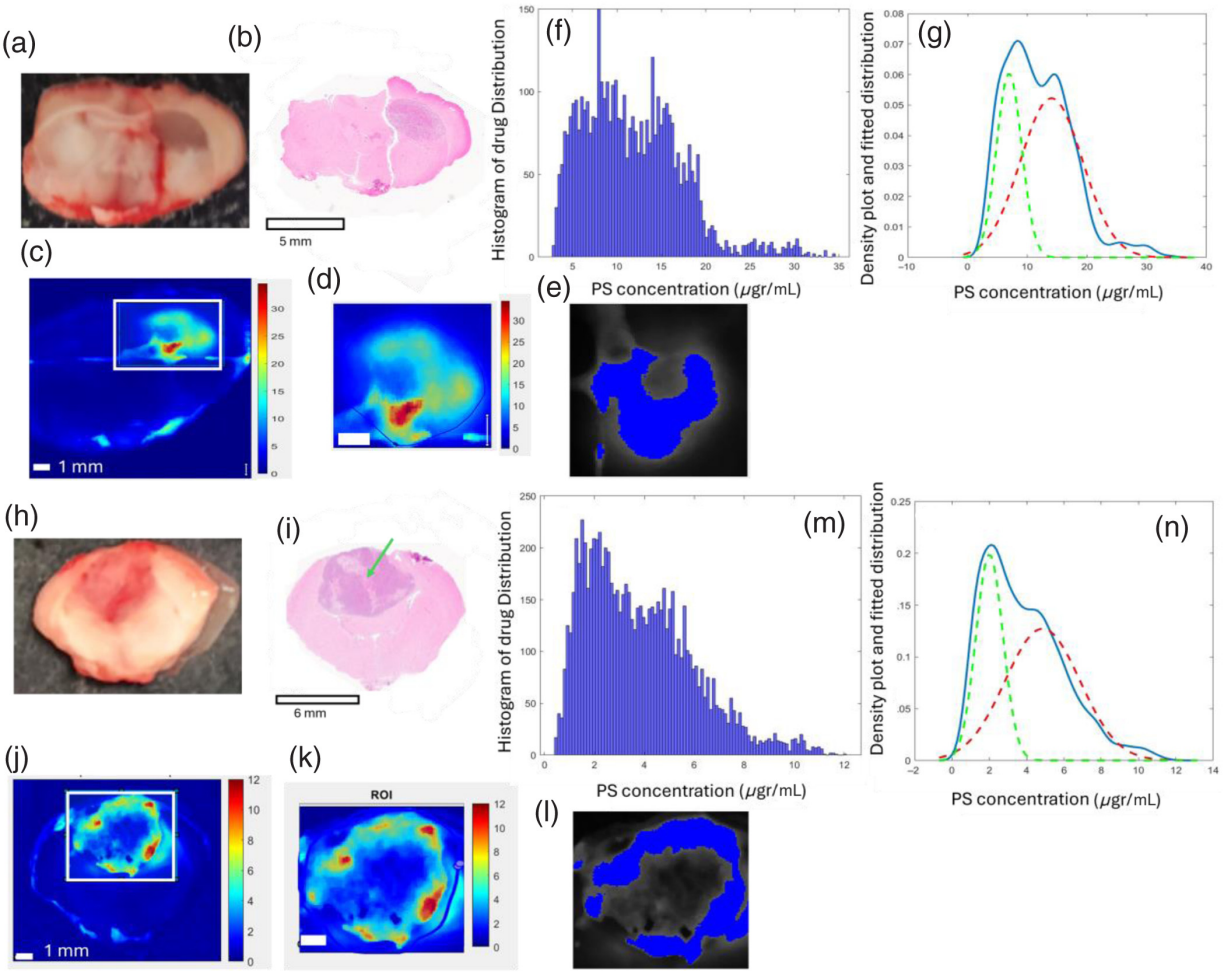
(a) Representative rat brain tissue slice from group A. (b) Histopathology of the slice section, identifying a tumor. (c) The porphysome fluorescence concentration map captured by qSFDI at $0.5\;{\mathrm{mm}}^{-1}$ corresponds to approximately the top 500-μm slice depth with a selected ROI encompassing the tumor. (d) Zoomed in tumor ROI. (e) Otsu threshold mask identified regions with an above-optimal threshold. (f) The corresponding frequency histogram within the tumor regions. (g) Density plot illustrating the spatial distribution of pixel intensities within the tumor, blue actual data, and green and red dotted lines fitted normal distributions with different. The same analysis is applied to the rat brain tissue slice from group B (Ce6 receiver), shown in the corresponding panels (h)–(n), following the same structure as panels (a)–(g). The color scale units are in $\mu g\;\mathrm{PS}\;{\mathrm{mL}}^{-1}$ tissue.

Figure 3 illustrates the qSFDI-derived distribution of [PS] at $0.5\;{\mathrm{mm}}^{-1}$ spatial frequency for both PSs in the brain slices of animals from groups A and B, along with histopathology slices, histograms, density plots, and their corresponding Otsu threshold masks.

The higher absolute PS concentration following the Porphysome administration versus Ce6 cannot be only due to the higher administered equivalent chlorin dose (5 versus $3\;\mathrm{mg}\cdot {\mathrm{kg}}^{-1}$) but may also be due to the enhanced permeability of the nanoparticle compared with molecular-based PS.

As reported by MacLaughlin et al.,^75^ 3% to 8% of the injected porphysome dose is present in the brain tumor 24 h post-administration. A porphysome dose of $10\;\mathrm{mg}\cdot {\mathrm{kg}}^{-1}$ equates to 60 to $160\;\mu g\cdot g^{-1}$ of tumor tissue for a rat weighing 200 g. Considering the total tumor weight, assuming that the density of $1\;g\cdot {\mathrm{cm}}^{-3}$ data indicates the total tumor uptake that the tumor uptake falls within the expected range.

The results depicted in Figs. 3(d) and 3(k) highlight the differential accumulation of porphysome and Ce6 within the tumor regions of groups A and B brain slices. The corresponding PS concentration histograms shown in Figs. 3(f) and 3(m) consistently demonstrate a non-normal distribution within the tumor regions for both groups, contrasting with the normal distribution typically observed in healthy brain regions. This non-normal distribution in the tumor areas can be modeled by two overlapping normal distributions with different modes, reflecting the heterogeneous and unpredictable nature of tumor vasculature across individual animals and among different PSs, as shown in Figs. 3(g) and 3(n).

This indicates that the [PS] distribution within the tumor regions exhibits two distinct subpopulations or distinct regions with varying central [PS] [see Figs. 3(e) and 3(l)]. The variation in uptakes among the animals is a function of tumor size (see Fig. S3 in the Supplementary Material). Otsu thresholding in larger tumors (diameter>2  mm) clearly distinguishes among regions, with higher concentrations mainly in the tumor rim area compared with lower concentrations in the tumor core area. Smaller tumors show less distinction between the two modes of distribution, indicating a more uniform uptake of PS across the tumor tissue. Higher PS accumulation in the outer rims compared with the tumor core was consistently observed in tumors larger than 2 mm in diameter.

**Fig. 4:**
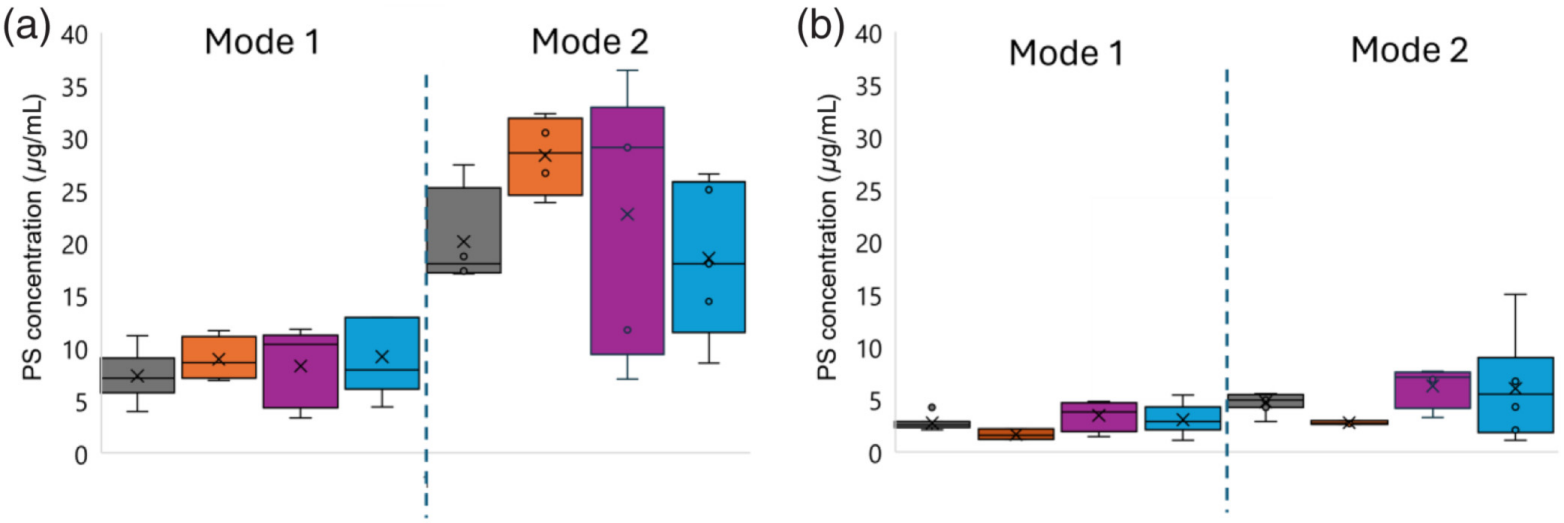
Boxplots of the two modes to fit the non-normal [PS] distribution across all slice sides for (a) Porphysome [PS] in four rats from group A and (b) for four rats from group B with Ce6 administration. Images are color coded for each rat as #1 (gray), #2 (orange), #3 (purple), and #4 (blue).

This is further supported by the box and whisker plots in Fig. 4, where the frequency histograms for all qSFDI images collected from rat tumors (21 slices from group A and 19 slices from group B) are fitted to two normal distributions with distinct modes. The bimodal distributions of PS concentrations show greater separation in larger tumors (diameter>2  mm) (rats 1 and 2 from group A, and rats 1, 3, and 4 from group B) compared with smaller tumors (diameter<2  mm) (rats 3 and 4 from group A, and rat 2 from group B), indicating increased heterogeneity in PS uptake with tumor size.

Although the concentration variation in mode 1 for both group A (porphysome receiver) and group B (Ce6 receiver) animals is small, the intra- and inter-rat variation of concentration in mode 2 was more pronounced as seen for rat 3 in group A, ranging from 7 to $36.42\;\mu g\cdot {\mathrm{mL}}^{-1}$ (5.2×) and rat 4 in group B from 2.60 to $15\;\mu g\cdot {\mathrm{mL}}^{-1}$ (5.7×).

The overall intra-tumor variation for the studied rats in both groups is summarized in Table S2 in the Supplementary Material. For group A, rats 2 and 3 exhibit the highest intra-tumor variability. For instance, rat 3 shows means and standard deviations of 8.26 and 2.62 for mode 1 and 22.74 and 5.41 for mode 2, corresponding to coefficient of variation (CV) values of 0.31 and 0.23, respectively. The interquartile range (${\mathrm{IQR}}_{10\%\text{ to }90\%}$) for drug concentrations in group A is $37.69\;\mu g\cdot {\mathrm{mL}}^{-1}$, indicating substantial dispersion.

In group B, rat 4 shows significant intra-tumor variability with means and standard deviations of 3.03 and 1.25 for mode 1 and 7.97 and 3.37 for mode 2, resulting in CV values of 0.41 and 0.42, respectively. The ${\mathrm{IQR}}_{10\%\text{ to }90\%}$ is $14.38\;\mu g\cdot {\mathrm{mL}}^{-1}$ within the group B.

Overall, group A exhibits greater intra-tumor variability, with higher mean and standard deviations in both modes than group B, indicating a more uneven drug distribution within the tumors.

#### 3.1.2 GLCM analysis

**Fig. 5:**
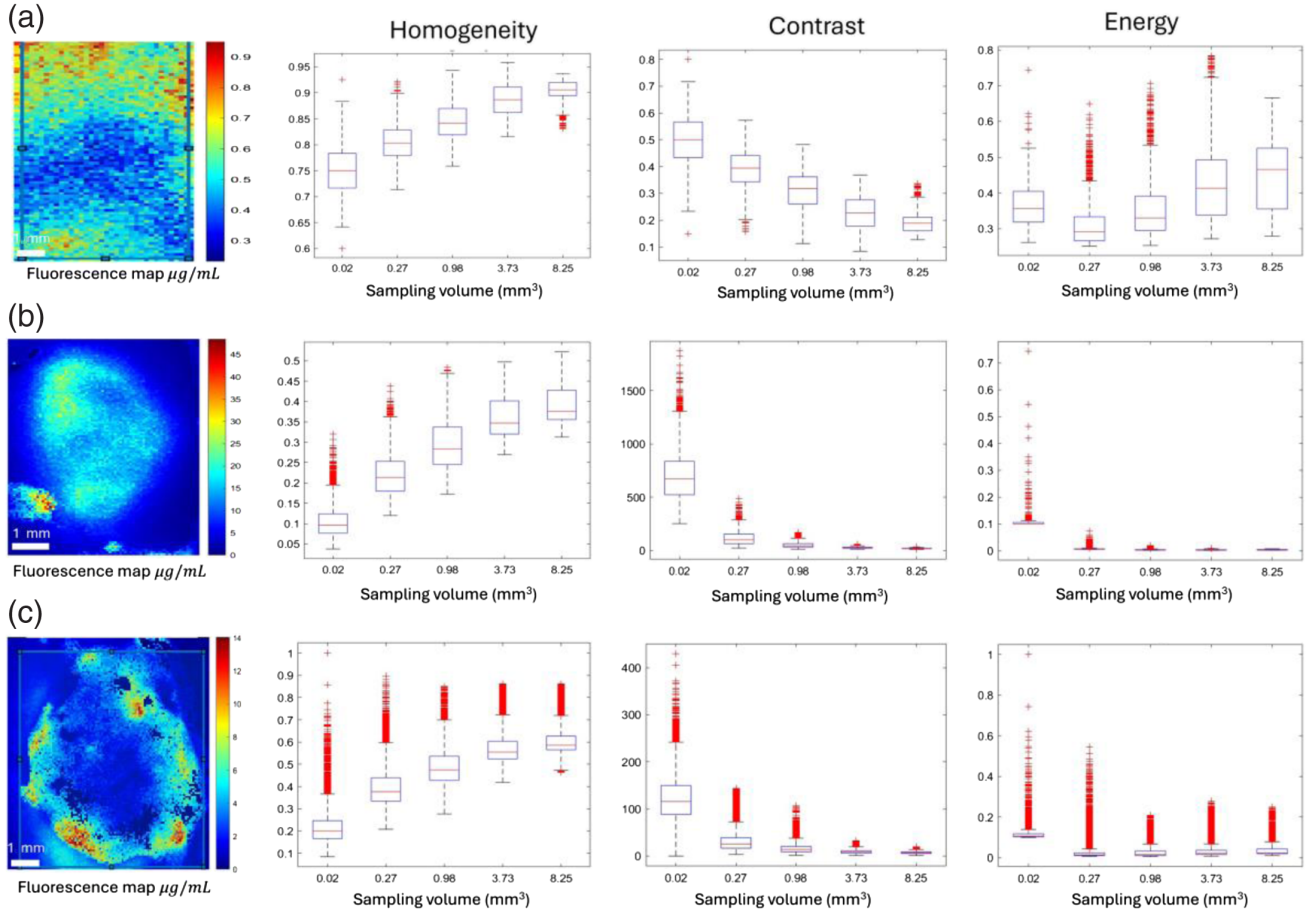
qSFDI fluorescence image captured at a spatial frequency of $0.5\;{\mathrm{mm}}^{-1}$, showcasing selected regions of interest (ROIs) along with homogeneity, contrast, and energy representing the GLCM texture analysis parameters computed within (a) a normal brain section, (b) a group A animal, and (c) from a group B animal. The selected scanning window, defined by a specific size, systematically traverses the ROI based on a defined step size. In this image, the sampling volumes range from a minimum of $0.02\;mm^{3}$ to a maximum of $8.25\;mm^{3}$.

Figure 5 illustrates a representative GLCM texture analysis for the three parameters of interest—homogeneity, energy, and contrast—for one ROI, each within normal and tumor from a brain slice in groups A and B animals.

For the normal contralateral brain, the [PS] varies in a very narrow range from 0 to $0.9\;\mu g\cdot {\mathrm{mL}}^{-1}$. The average homogeneity is close to 0.75 with the smallest sampling volume and increases with the sampling volume, approaching 1. The contrast is highest at the smallest window size and decreases as the window size (i.e., sampling volume) increases, approaching 0. This indicates a relatively small variation in patterns of homogeneous fluorescence emission and, hence, [PS]. Energy also increases with window size, reflecting uniformity and echoing the observations of homogeneity and contrast.

Fluorescence emission from tumors exhibits a much lower homogeneity at the highest resolution and increases with the expansion of the window size. Conversely, contrast is higher at the smallest sampling volume ($0.02\;mm^{3}$), indicating significant intensity variation at finer resolutions, and decreases with larger window sizes, reflecting less variation. However, energy values are lower for tumor regions than normal brain tissue, suggesting greater texture variability and lower orderliness. These values decrease as the window size grows, indicating less uniform textures.

Figure 5 highlights heterogeneity likely exists at the microscale, specifically at scales less than 200  μm. At a 3×3  pixel window size, equivalent to $0.02\;mm^{3}$, a significant portion of this heterogeneity, ∼90%, is captured. This is derived from the observation that homogeneity is around 10%, thus implying that heterogeneity (1-homogeneity) is ∼90%. This fine resolution captures the detailed texture variations within the tumor. However, whether determining heterogeneity at high spatial resolution will impact PDT treatment planning or if lower resolution is sufficient needs to be established. Therefore, one must understand what imaging systems can provide sufficient resolution when deciding on the appropriate scale for analysis in our study.

Section 3.2 uses generated simulation models with different spatial resolutions captured by GLCM analysis to determine the spatial scale of heterogeneity that influences the PDT-SPACE treatment planning solution.

### 3.2 Impact of PS Heterogeneity on iPDT Treatment Planning Solution

**Fig. 6:**
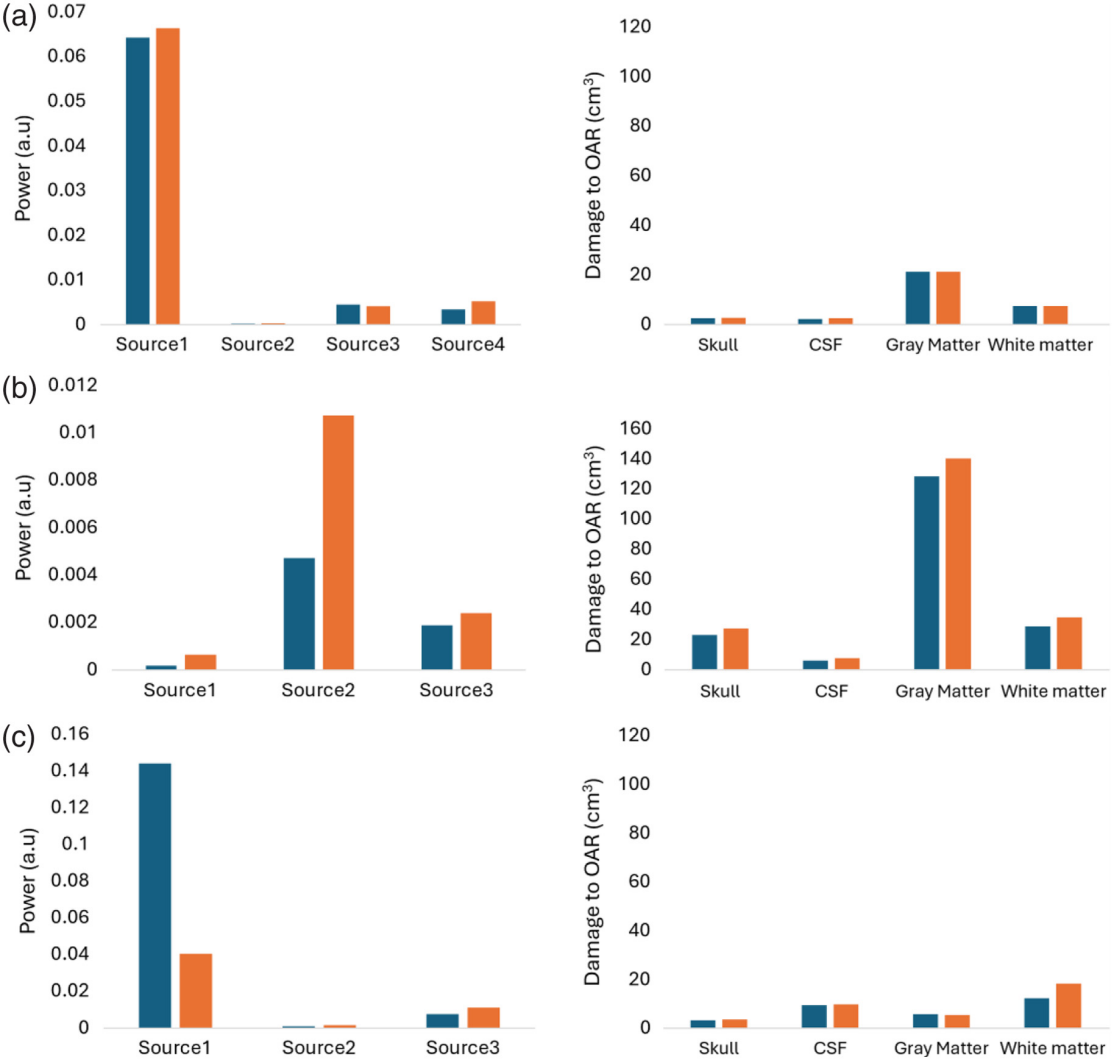
(a) PDT-SPACE results for a high spatial resolution model. (Left) The power allocations for sources (1 to 4) at fixed source positions with homogeneous (blue) and heterogeneous (orange) PS distributions. (Right) The potential damage to OAR (in $cm^{3}$) when 98% of the tumor is destroyed under the same conditions. (b) PDT-SPACE results for a low spatial resolution model. (Left) As described above, the source power allocations and (right) the potential damage to OAR (in $cm^{3}$). (c) PDT-SPACE results for a non-spherical medium spatial resolution model with the same left and right components.

The first part of the simulation study aimed at studying the difference in the PDT-SPACE-derived treatment protocol for homogenous versus heterogenous [PS] in the target volume for three high- and three low-resolution brain tumor cases. Figure 6 shows the PDT-SPACE planning solutions for optical power delivery in one of high-, medium- and low-resolution simulations using fixed source positions. The PDT-SPACE outcome also provides the tissue volumes at risk for the adjacent brain structures. The results for the remaining high spatial resolution models are shown in Fig. S4 in the Supplementary Material, whereas the results for the low-resolution models are shown in Fig. S5 in the Supplementary Material.

Interestingly, the findings indicate that PDT-SPACE generates similar solutions regarding required power for the interstitial sources in homogeneous and heterogeneous cases for all three high-resolution meshes. The tissue volumes at risk for the OARs, given in cubic centimeters, were similar for all three cases.

However, for the low spatial resolution models, notable differences in the PDT-SPACE solution between homogeneous and heterogeneous cases became apparent. In addition, significant differences in OAR damage were observed. A possible limitation here is the above-mentioned CV observed by Wang et al., which could impact the actual numerical results; however, this will not affect the general conclusion.

Furthermore, the simulation was run for both spherical and non-spherical medium-resolution models, as shown in Figs. 2(d) and 2(e), to determine the sensitivity of the PDT-SPACE program to medium spatially resolved photosensitizer concentrations. The results showed significant differences in power and damage observed between the homogeneous and heterogeneous models for both, indicating that PDT-SPACE is sensitive to variations at this scale. Figure 6(c) shows the results for a non-spherical medium-resolution model.

The results shown in Fig. 6 indicate that, with fixed source positions and only power optimization, there was relatively high damage to the OARs for both homogeneous and heterogeneous models. The PDT-SPACE simulated annealing (SA) engine was run for low-resolution models to exploit all planning options by moving the sources multiple times to reduce OAR damage.

**Fig. 7:**
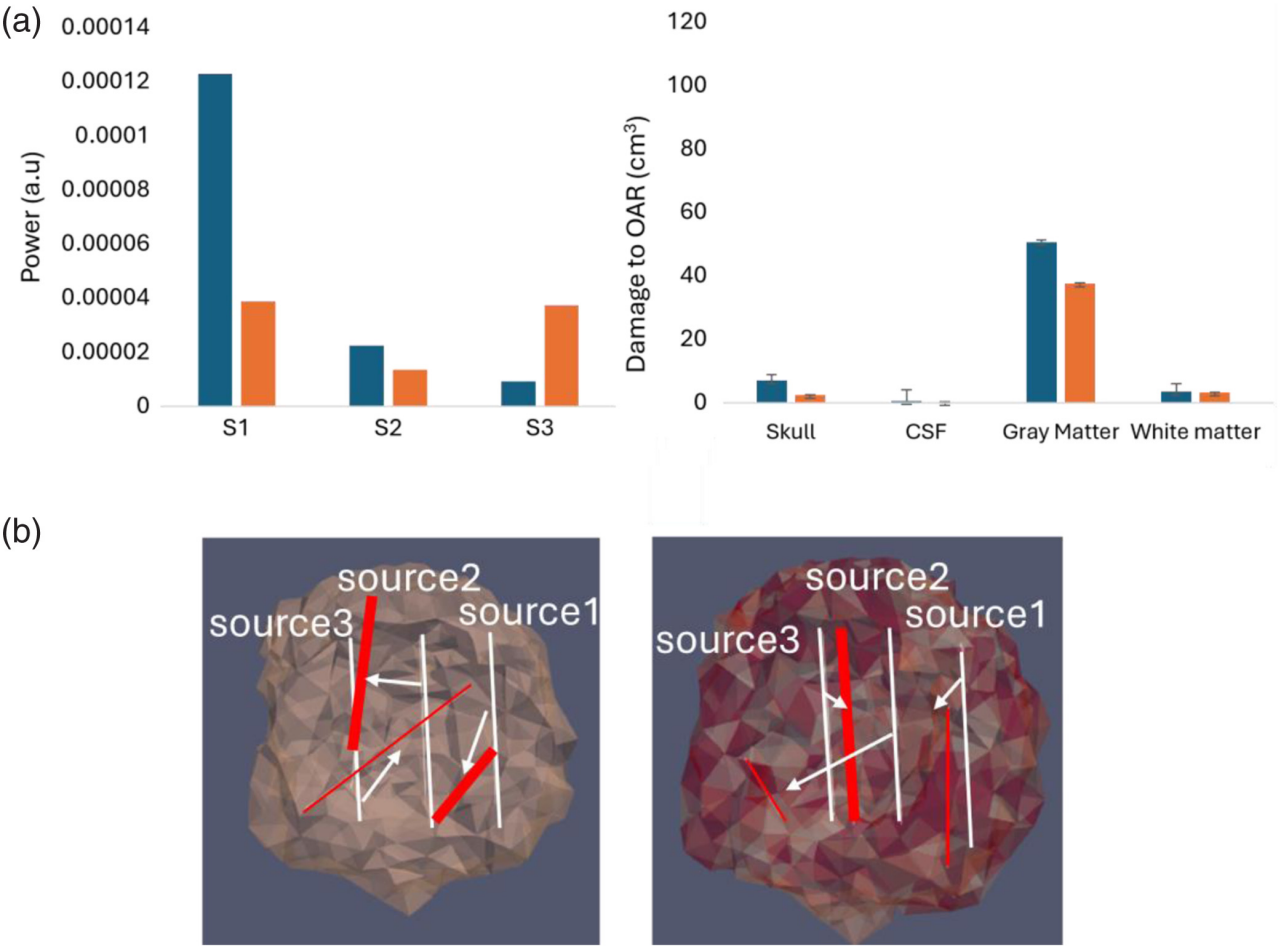
(a) PDT-SPACE results for the same low spatial resolution model: (Left) Power allocations for sources 1 to 3 using a simulated annealing (SA) engine with homogeneous (blue) and heterogeneous (orange) PS distributions; (right) the resulting potential damage to OAR (in $cm^{3}$) when 98% of the tumor is destroyed under the same conditions. The error bars represent the variation in the results due to the use of a random seed in the SA simulations. (b) The initial (white) and final (red) source length and positions for homogeneous (left) and heterogeneous (right) PS distributions. The increased line thickness corresponds to the power gain after optimization.

Figure 7 shows the PDT-SPACE results for source power and potential damage to OARs and compares the final source length and positions (shown in red) to the initial ones (shown in white). When optimizing for source length and positions, the damage to OAR was significantly reduced. In addition, heterogeneity further reduced the damage.

Having established that PDT-SPACE power allocation can impact PDT performance considering the spatial distribution of [PS] at a certain spatial resolution, PDT-SPACE was executed for the four models of scenario 3 where different [PS] distribution patterns were generated within the tumor rim, comparable to real-life scenarios observed *in vivo*.

The second part of the simulation aims to examine the variations among the three scenarios outlined in Sec. 2.3. Specifically, it compares the power allocation solutions and the differential damage to the OAR.

**Fig. 8:**
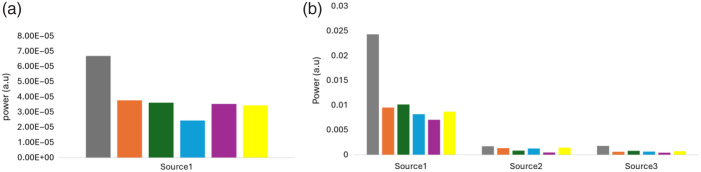
Comparison of PDT-SPACE source power allocation for a tumor in scenario 1 (gray), scenario 2 (orange), and the four models of scenario 3 with model 1 (green), model 2 (blue), model 3 (purple), and model 4 (yellow) for (a) spherical tumors utilizing a single source (S1) and (b) non-spherical tumors utilizing three sources (S1 to S3).

Figure 8 illustrates the PDT-SPACE power allocation for both spherical and non-spherical tumors. A single light source was sufficient for spherical tumors, with zero power assigned to sources 2 and 3, whereas non-spherical tumors required three light sources for adequate coverage. The figure also compares the four models from scenario 3 with results from the homogeneous [PS] case (scenario 1) and the homogeneous [PS] case with differences between the tumor core and rim (scenario 2). The analysis shows the highest power demand in scenario 1, with comparatively lower power requirements in scenarios 2 and 3.

**Fig. 9:**
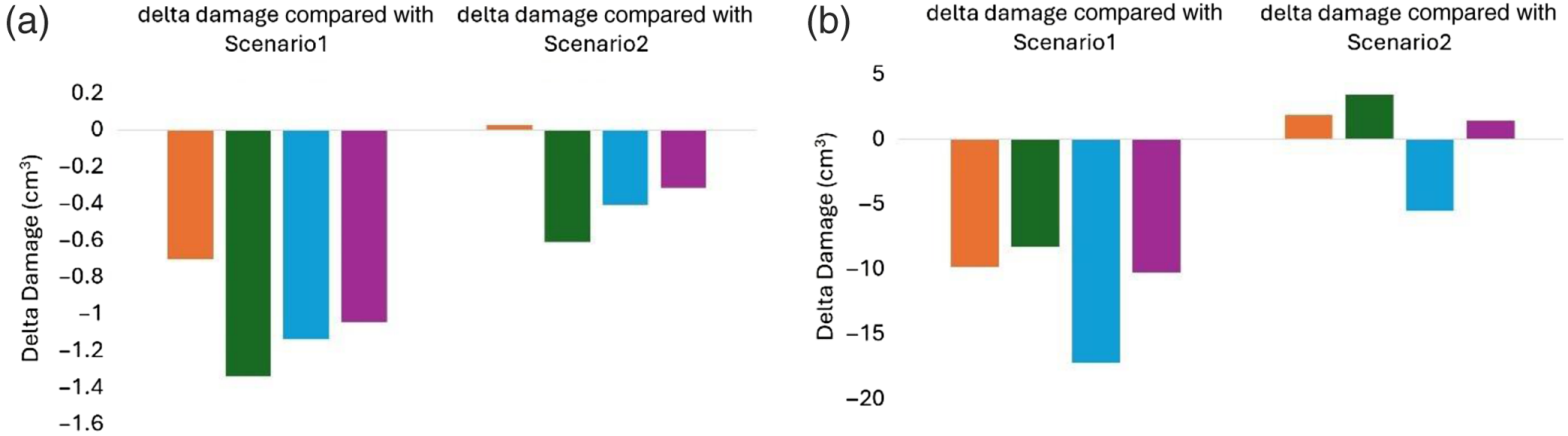
Delta damage (${\mathrm{cm}}^{3}$) to gray matter (GM) among four models of scenario 3, model 1 (orange), model 2 (green), model 3 (blue), and model 4 (purple), with scenario 1 on the left and scenario 2 on the right, for (a) the spherical models (b) non-spherical models when using fixed source positions.

PDT-SPACE power allocations are the highest when assuming homogenous [PS] in both spherical and non-spherical models. Power allocation reduction is primarily achieved by separating the [PS] in the core and rim; however, the actual power allocation in the four models of scenario 3 was still dependent on the particular PS distribution, shown in Fig. S2 in the Supplementary Material. Reducing the power allocations for scenarios 2 and 3 simulations generally had the benefit of reducing the OAR volume for almost all spherical models, as shown in Fig. 9(a). This is evident when comparing the delta damage for four models of scenario 3 to the homogeneous tumor in scenario 1 or versus scenario 2 with homogeneous but different [PS] in the tumor core and rim.

For the gray matter (GM) volume at risk, the reduction ranged from 0.7 to $1.34\;cm^{3}$ for the spherical tumor when accounting for heterogeneity in the planning process. The reduction in the OAR volumes was more modest compared to scenario 2 but could reach $0.6\;{\mathrm{cm}}^{3}$.

Figure 9(b) shows that the four [PS] distribution models in the non-spherical tumor of scenario 3 also demonstrated reduced damage in volumes of the OAR, ranging from ∼8 to $17\;cm^{3}$, compared with scenario 1. However, no clear gains in reducing the OAR damage volume are evident for the four heterogeneous models compared with scenario 2 in non-spherical models.

The PDT-SPACE-simulated annealing (SA) engine was run for both spherical and non-spherical models across all three scenarios to optimize the PDT-SPACE solution with regard to the light source positions, source power, and potential delta damage. The SA engine calculated the optimized source positions for all scenarios, and the total displacement for each source was determined using Euclidean distances between centers. Table 2 summarizes the displacement comparison for each source among different scenarios in non-spherical models.

**Table 2:** Displacement comparison of source positions across different non-spherical models and scenarios.

Source displacement	Source 1 (mm)	Source 2 (mm)	Source 3 (mm)	Source 4 (mm)
Scenario 1 versus scenario 2	1.05	9.33	15.34	11
Scenario 1 versus scenario 3 model 1	4.74	6.87	25.79	22.21
Scenario 1 versus scenario 3 model 2	2.05	6.25	5.8	1.86
Scenario 1 versus scenario 3 model 3	7.91	10.95	11.13	16.66
Scenario 1 versus scenario 3 model 4	4	7.93	18.33	17.22
Scenario 2 versus scenario 3 model 1	4.51	3.04	20.84	19.91
Scenario 2 versus scenario 3 model 2	3.09	4.47	15.57	10.64
Scenario 2 versus scenario 3 model 3	8.87	12.03	11.65	12.78
Scenario 2 versus scenario 3 model 4	3.28	9.16	10.88	11.24

Comparing the source displacement between scenarios 1 and 2, a notable displacement up to ∼15  mm in source 3 highlights significant differences when only a lower [PS] in the core is considered.

Displacement for all four sources across is generally more significant for all scenarios, three models versus scenario 1 than scenario 2. The individual source displacements in the different models suggest the sensitivity of the PDT-SPACE planning tool to particular heterogeneous patterns. The displacement comparison across different scenarios for spherical models is shown in Table S2 in the Supplementary Material. For the spherical models, similar to the non-spherical models, we observed significant displacements in source positions, indicating the impact of varying [PS] distributions on treatment planning and effectiveness. A comparison between the source power is not applicable as this is calculated with respect to the position of the sources, which varies for all models.

**Fig. 10:**
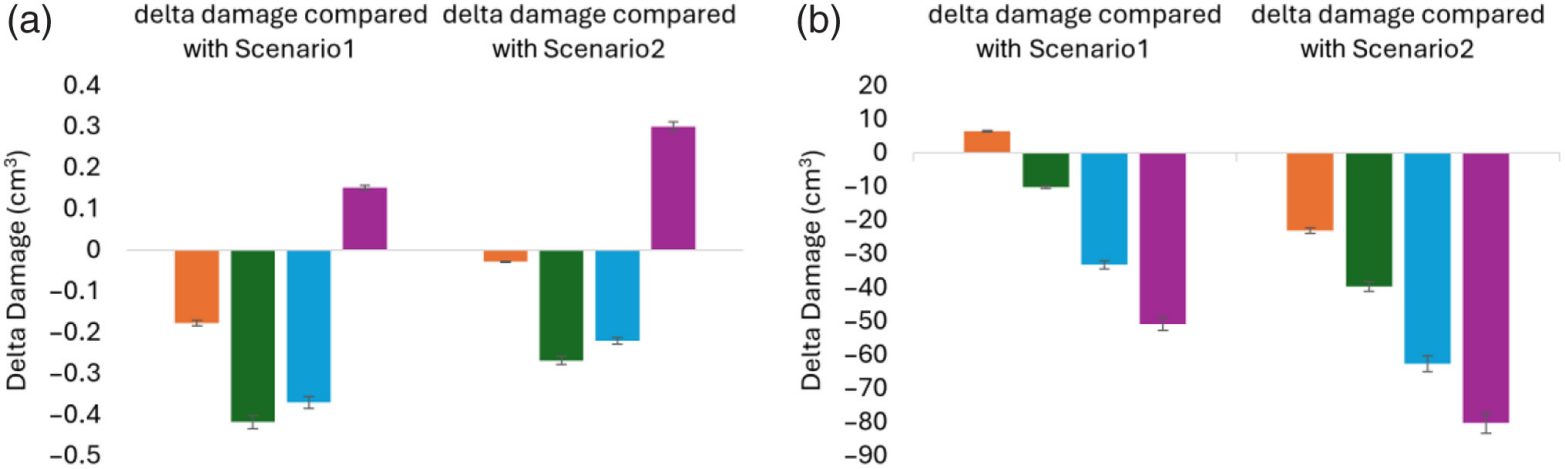
Delta damage (${\mathrm{cm}}^{3}$) to gray matter (GM) among four models of scenario 3, model 1 (orange), model 2 (green), model 3 (blue), and model 4 (purple), with scenario 1 on the left and scenario 2 on the right, for (a) the spherical models (b) non-spherical models after optimizing for source placements using SA engine. The error bars represent the variation in the results due to using a random seed in the SA simulations.

Figure 10(a) compares the delta damage for all four spherical models of scenario 3 to scenario 1 on the left and scenario 2 on the right. There was a minor reduction in damage to gray matter for all models except for model 4, where maintaining 98% tumor damage, the OAR volume increased. In the non-spherical tumor models [Fig. 10(b)], the reductions in gray matter volume at risk were more pronounced. The reduced damage ranged from 10 to $50\;cm^{3}$ compared with scenario 1 and from 23 to $80\;cm^{3}$ compared with scenario 2.

## 4 Discussion

The majority of iPDT dosimetry studies and clinical translation focus on monitoring the progress of the delivered dose following standardized placement of the treatment light sources in the CTV. At this time, the physician or surgeon can only modify the rate of power delivery, including intermittent illumination breaks and the total energy delivered, independent of the surface illumination and interstitial delivery. Zhou et al.^11^ indicated that tumors of the same type exhibit significantly diverse microscopic distributions of PS given the time required for drug extravasation from the higher intravascular [PS] to distant sites of the tumor epithelium. Fiber optics probes have been used to quantify [PS] locally; however, given its clearance rate and the associated changes to the spatial concentration gradient, quantification measurement of the drug concentration at early times will always be confounded by this compartmentalization issue.^10^^,^^11^ In addition, the minimal sample volumes for interstitial fiber probes are on the order of several cubic millimeters and larger, given the probe size and median tissue scattering distance. Finally, achieving information about [PS] variability requires the insertion of multiple sensor fibers; however, this is not feasible for all organ tissue.

In previous studies,^36^^–^^39^^,^^43^^–^^45^ we demonstrated that pre-treatment planning of interstitial light source placement and optimizing the power or energy delivered to each source can reduce the volume of CTV adjacent tissue at risk for PDT-induced damage while maintaining the fluence sufficiently high within the CTV so that at least 98% of its volume receives a lethal PDT dose. The PDT-SPACE optimization process currently requires knowledge of the tumor’s and OAR’s average homogeneous [PS] as well as their responsivity to PDT. However, the assumption of homogeneous [PS] is incorrect, as demonstrated by Refs. 11, 24–26, and 28. Spatial variations in [PS] occur at macroscopic and microscopic scales, and the variance in [PS] can depend on the spatial resolution of the imaging system.^11^ While incorporating spatial [PS] variability in the treatment planning software, a key question to be answered is at what spatial resolution the [PS] variability needs to be known to result in a different PDT planning solution.

To the best of our knowledge, this is the first study to employ *in silico* simulation results to determine the desired resolution at which [PS] variations can impact PDT treatment outcomes. *In vivo*, preclinical studies were performed to match *in silico* simulations to biological and potentially clinically relevant [PS].

All qSFDI image analyses showed a non-normal [PS] distribution, as shown in Fig. 4. The qSFDI-derived histogram revealed a considerable inter-tumor variation in PSs, with the maximum 10th to 90th percentile interquartile range for drug concentrations being $37.69\;\mu g\;{\mathrm{mL}}^{-1}$ for porphysome within group A animals and $14.38\;\mu g\cdot {\mathrm{mL}}^{-1}$ for Ce6 within group B animals at a spatial frequency of $0.5\;{\mathrm{mm}}^{-1}$.

Integrating the spatial [PS] over the tumor volume resulted in a total uptake comparable to that in the literature.^75^ The uptake values for porphysomes are higher than for Ce6, possibly due to the enhanced permeability of the nanoparticles^48^ compared with the molecular version of the active PS.

In addition, two spatial regions with distinct PS accumulation were observed, coinciding with the tumor’s necrotic and poorly perfused cores and the highly metabolic active and well-perfused tumor rims, as shown in Fig. 3 and Fig. S3 in the Supplementary Material. Thus, the [PS] in the rim appears to have a higher uptake than suggested by bulk extraction techniques, whereas the [PS] in the core is significantly lower. This is consistent with the work of Elliott et al.,^12^ where they showed enhanced blood flow and blood volume in the tumor rim area and a decrease in the core. This large difference in the [PS] between these two regions is often ignored despite being well documented for chemo- and photodynamic-therapy. Although the PS uptake and retention variabilities in the core and rim can be modeled with normal distributions, the mode of the normal distribution shows variability within and between animals and for the two PSs. The underlying factors contributing to the inter-tumor heterogeneity in PS uptake and retention are not fully elucidated. However, it is evident that the irregular vascular patterns within tumors create a highly heterogeneous and unpredictable microenvironment.^76^ Furthermore, it was indicated that tumor size also impacts the scale of heterogeneity in a way that larger tumors showed more separation between the two modes of distribution. This is likely because of a larger necrotic core and damaged blood vessels. This variation directly impacts PDT dosing as the generation of reactive oxygen species (ROS) is influenced by [PS]. In group A, with a higher and more variable mode 2 concentration, ROS generation could be significantly higher, requiring careful adjustment of PDT doses to avoid excessive damage to surrounding healthy tissues. Conversely, group B’s lower and less variable mode 2 concentration suggests a more predictable but potentially less effective ROS generation, which might necessitate higher PDT doses to achieve the desired therapeutic effect.

Our GLCM texture analysis illustrated that the distribution observed with smaller sampling is wider than that observed when sampling is done over larger tissue areas. Thus, sampling fluorescence from larger and larger regions has the effect of averaging the distribution function toward the mean value, and there is a loss of the lowest and highest parts of the histogram. Thus, pixel-level sampling volume was determined as the minimum scale over which the tumor’s highest heterogeneity can be captured. Heterogeneity exists at the microscale, <200  μm, probably stemming from intra-capillary spacings. At a 3×3  pixels window size, equivalent to $0.02\;{\mathrm{mm}}^{3}$, a significant portion, 90%, of this heterogeneity is captured.

A simulation study was conducted to determine whether microscale heterogeneity impacts the treatment planning solution and potentially affects the treatment outcome. In other words, the study aimed to identify the minimum spatial scale or resolution—that is, the sampling size—at which the impact on PDT treatment planning solutions becomes noticeable. Furthermore, we examined the effects of accounting for heterogeneity in PDT treatment planning across various heterogeneity scenarios.

Our simulation results, however, indicated that capturing heterogeneity across sampling volumes at a high resolution of $0.02\;{\mathrm{mm}}^{3}$ is not required as no changes between the PDT-SPACE treatment planning solutions were noted between the homogeneous and heterogeneous models. This is due to the optical properties determining the fluence gradient and, hence, the spatial light distribution. Light source placements and power allocations cannot compensate for PDT dose variabilities at spatial domains smaller than the inverse of the tissues’ effective attenuation coefficient. However, modeling heterogeneity at median resolutions of $∼1.05\;{\mathrm{mm}}^{3}$, a scale attainable with current clinical imaging, resulted in distinct treatment plans between the homogeneous and individual heterogeneous models.

Looking at various heterogeneous models with different patterns of heterogeneity comparable to real tumor heterogeneity for both spherical and non-spherical models, it was identified that the proposed treatment plan by PDT-SPACE varies significantly across different scenarios: scenario 1 (homogeneous [PS] throughout the entire tumor, including the core and rim), scenario 2 (homogeneous [PS] but differing between the core and rim), and all four models of scenario 3 (models 1 to 4), where heterogeneous [PS] is added to the rim, in addition to low [PS] in the core. The damage to OAR also varied, with mostly less damage observed when considering a lower concentration in the core than in the rim compared with the same concentration in the core and rim. Considering heterogeneity in the core and rim in addition to lower [PS], the damage to OAR varied depending on the pattern of heterogeneity. The power is calculated to maintain 98% damage to the tumor while causing minimum damage to healthy OAR. Thus, depending on heterogeneity patterns, certain models exhibit increased organ damage to maintain 98% damage to the tumor.

For some scenarios, the mean value of [PS] may be sufficient. However, there are cases when considering the PS’s heterogeneity can significantly reduce the damage to the OAR and thus improve the treatment outcome. These results highlight that the non-spherical models consistently showed significantly reduced volumes in the GM at risk, suggesting a more significant benefit of accounting for heterogeneity in these cases.

The source placement analysis also showed significant positional adjustments of sources across scenarios, highlighting the sensitivity of PDT to spatial variation in [PS] and the need for careful optimization of source positions in PDT to ensure effective treatment, particularly with heterogeneous [PS] distributions.

This study highlights the potential variation in PDT dose due to poor planning concerning only homogeneous average PS concentration, which in some cases results in a patchy PDT response and treatment heterogeneity, as shown by Weersink et al. in the case of prostate where a low fluence rate was used.^77^^,^^78^ A large degree of variation in [PS] is problematic, especially when no PS dosimetry is implemented, and every subject receives the same stipulated light irradiation. Zhou et al.^11^ indicated that accurate quantification of tissue PS levels and subsequent adjustment of the light dose would allow for reduced subject variation and improved treatment consistency. However, there are situations where the patient does not accumulate enough [PS] in the tumor due to many less well-perfused vessels and a large necrotic area. Subjects with lower PS uptake could be undertreated, resulting in partial response or even no response to the PDT treatment or potentially even worse complications such as enhanced aggressiveness of the disease.^11^

Thus, predicting the spatial [PS] prior to treatment is necessary to determine patient eligibility and to plan pre-treatment considering the variation in [PS]. Pre-treatment planning also determines whether measuring the lowest and highest [PS] is important or useful for predicting tumor response or if the average [PS] will suffice.

This study’s important observation is that spatial [PS] can serve as a valuable metric of treatment efficacy. It is most effective when employed prior to treatment to determine whether the patient is eligible for PDT and to adjust the total dose required for treatment on an individual-subject basis. The current real-time PS dosimeters designed for online, real-time measurement of [PS] in tumors could be used solely for real-time treatment planning methodology and for optimum placement of the treatment light sources.

## 5 Limitations

Significant variability in PS uptake by tumors has been observed in both animal and human studies.^9^^,^^79^ Zhou et al. revealed that the variation in PS uptake within a single tumor is unpredictable and does not correlate with the differences observed between separate tumors.^9^^,^^11^ Our study, focusing on a single animal tumor type and two distinct drugs, highlights the potential for different tumors to exhibit varying degrees of heterogeneity. This is due to differences in drug uptake and the tumor microenvironment, necessitating the individual determination and quantification of this heterogeneity for each specific tumor, which presents a formidable challenge.^11^

Studying tumor heterogeneity in humans is challenging due to limitations in biopsy sampling, which also would not fully capture the tumors’ spatial and temporal diversity. Although genetically engineered mouse models provide sophisticated means of modeling human cancer development and allow for longitudinal studies in a controlled environment, their limited genetic heterogeneity and reproducible growth fail to capture the full variability in drug uptake.^80^ In this study, we used a rat model to demonstrate the effect of heterogeneity on the PDT-SPACE pre-treatment planning program, highlighting the need for personalized treatment planning that considers heterogeneity in [PS]. It is important to note that the scale of this heterogeneity in animal models and humans might not be the same. Still, our findings emphasize the critical role of heterogeneity in effective treatment planning.

It is worth mentioning that other factors besides PS concentration will affect PDT treatment efficacy, including oxygen availability and cellular response within different microenvironments present in the tumor. In this study, we focused solely on heterogeneity in [PS] as the primary factor determining efficacy. Assessing tumor oxygenation at the spatial resolutions indicated above is necessary for more effective planning. Hence, predicting PDT outcomes following personalized photon fluence distribution planning is the first step in exploiting the therapy’s full potential for interstitial tumor destruction.

## 6 Conclusion

There are considerable intra- and inter-tumor variations in [PS] uptake. The magnitude of the variations appears to be PS-specific. The PS variation between individual tumors leads to a variation in treatment response after PDT treatment.

Despite the lack of predictability in the heterogeneity pattern observed, a consistent trend emerges wherein lower PS concentrations are found in the tumor core, attributed to limited vascular perfusion compared with the tumor’s rim, presenting as a highly perfused area. Although this heterogeneity manifests on macroscopic and microscopic scales, our simulation results indicate that treatment planning considering the PS heterogeneity is required at a minimum spatial scale. This study determined and considered the spatial resolution to which the heterogeneity impacts the PDT-SPACE resolution as a function of the [PS] variance in the PDT-SPACE treatment planning process. We demonstrated that determining [PS] at resolutions achievable with clinical imaging systems, such as MRI or CT, can significantly impact PDT-SPACE’s solutions for PDT treatment planning. This could lead to clinically significant modifications in treatment plans and potential improvements in treatment outcomes. We also demonstrated that sampling [PS] at very high resolutions, comparable to capillary spacing, is not required, as the treatment plan will not differ between homogeneous and heterogeneous PS distributions at those very high spatial resolutions. However, this conclusion is based on the specific resolutions tested, and further investigation with additional resolutions is needed to confirm the minimum spatial resolution necessary for accurate determination of Δ[PS]. In addition, various scenarios of heterogeneity, comparable to observed heterogeneity in animal models, were simulated, and the variations in PDT-SPACE solutions and potential damage to OAR were compared. Attaining a clinically significant reduction in damage to the hypothetical OAR was noted when considering variation in the local [PS] compared with the population average.

The minimum number of required light sources can be found by determining the optimized cylindrical diffuser’s positions, lengths, and power allocations prior to treatment while retaining the desired outcome and avoiding mechanical damage due to fiber insertion within the tumor.

Studying intertumoral heterogeneity through random sampling or biopsy is difficult and fails to capture the complete range of phenotypic or genetic diversity within a tumor. Therefore, a non-invasive approach to evaluate tumor heterogeneity could be clinically valuable, especially in the era of personalized medicine, by identifying patients with poor prognoses who may benefit from more intensive therapy. Consequently, tumor heterogeneity is a clinically significant parameter for imaging, potentially quantifiable, and could enhance standard reporting methods.^81^

To achieve this, our study advocates for innovative surrogate techniques to predict the [PS] using available clinical imaging systems such as MRI as the resolution is adequate for effective heterogeneity measurements, thereby improving pre-treatment planning. This can lead to an increased understanding of the underlying mechanisms of heterogeneous distribution and facilitate patient selection for PDT. This helps to assess potential therapy-associated risks and prevents patients from being deprived of the opportunity to seek other potentially more suitable therapeutic approaches.

## 7 Future Work

Blood flow (BF) and blood volume (BV) are shown to be correlated with PS biodistribution in tumors. Using the VX2 tumor model in rabbit pancreas and the vascular-acting PS Verteporfin, Elliott et al.^12^ proposed a linear correlation between the PS’s fluorescence *ex vivo*, and BF, BV, and the permeability surface-area product. For cellular-acting PSs, where the extravasation and diffusion coefficient of the PS play a significant role in tissue drug accumulation, Jespersen and Østergaard^82^ introduced the concept of mean transit time (MTT) as a predictive tool. They demonstrated the applicability of MTT, defined as the ratio of BV to BF, to predict the maximum extraction of small molecules. In future studies, we aim to utilize MRI to estimate these parameters and contribute to a deeper understanding of the dynamics between blood perfusion and PS distribution in tissue. This approach holds promise for advancing our knowledge in the context of predicting spatial [PS] and implementing it into PDT-SPACE to improve personalized PDT pre-treatment planning.

## Supplementary Material



## Data Availability

All the FullMonte tools, including FullMonteWeb, are available at http://FullMonte.org/. The source code for the tools and all meshes used in the paper are available at https://gitlab.com/FullMonte.
