# Supplementary material for: Photosensitizer spatial heterogeneity and its impact on personalized interstitial photodynamic therapy treatment planning
Source: J Biomed Opt. 2025 Jan 11;30(1):018001. doi: 10.1117/1.JBO.30.1.018001 (PMC11724368; doi:10.1117/1.JBO.30.1.018001)
Supplement: Supplementary file 1 [file JBO_030_018001_SD001.pdf]

Appendix A: Supplementary materials

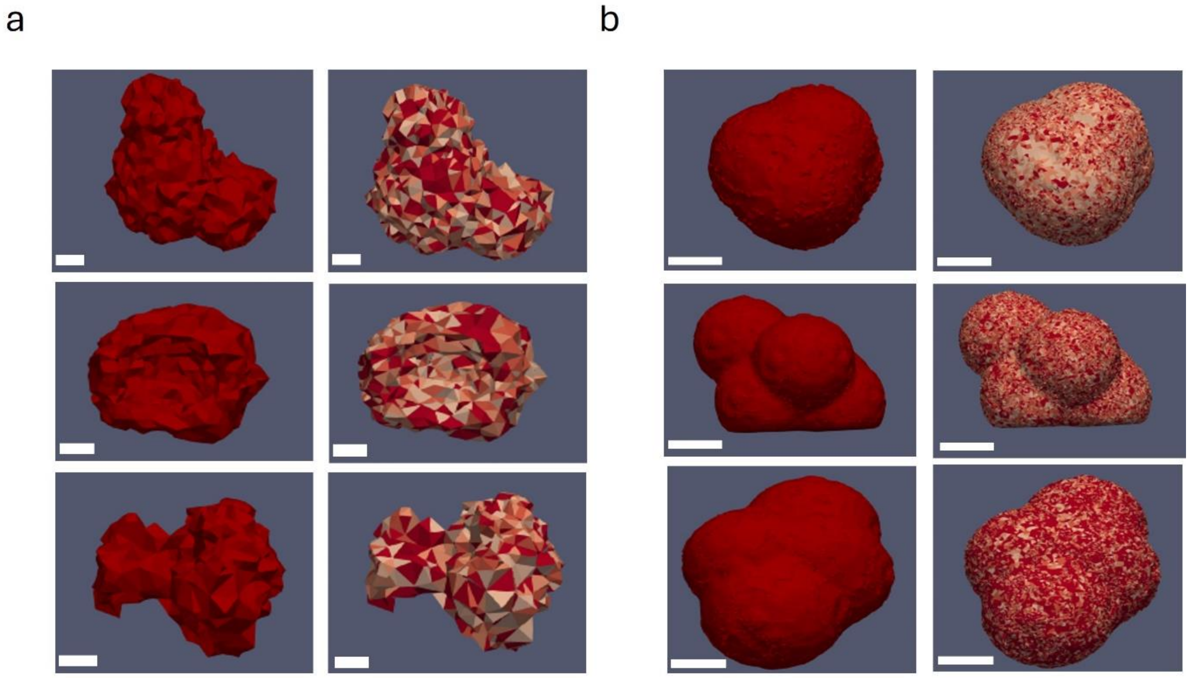

Fig.S1 (a) Three low-resolution tumor models of different shapes and sizes with an average tetra size of 2-3 mm3, showing homogeneous [PS] (left) and heterogeneous [PS] (right). **(b)** Three high-resolution tumor models of different shapes and sizes with an average tetra size of 0.02 mm3, showing homogeneous [PS] (left) and heterogeneous [PS] (right). The average [PS] in all heterogeneous models is equal to the homogeneous models. White scale bars correspond to 20 mm.

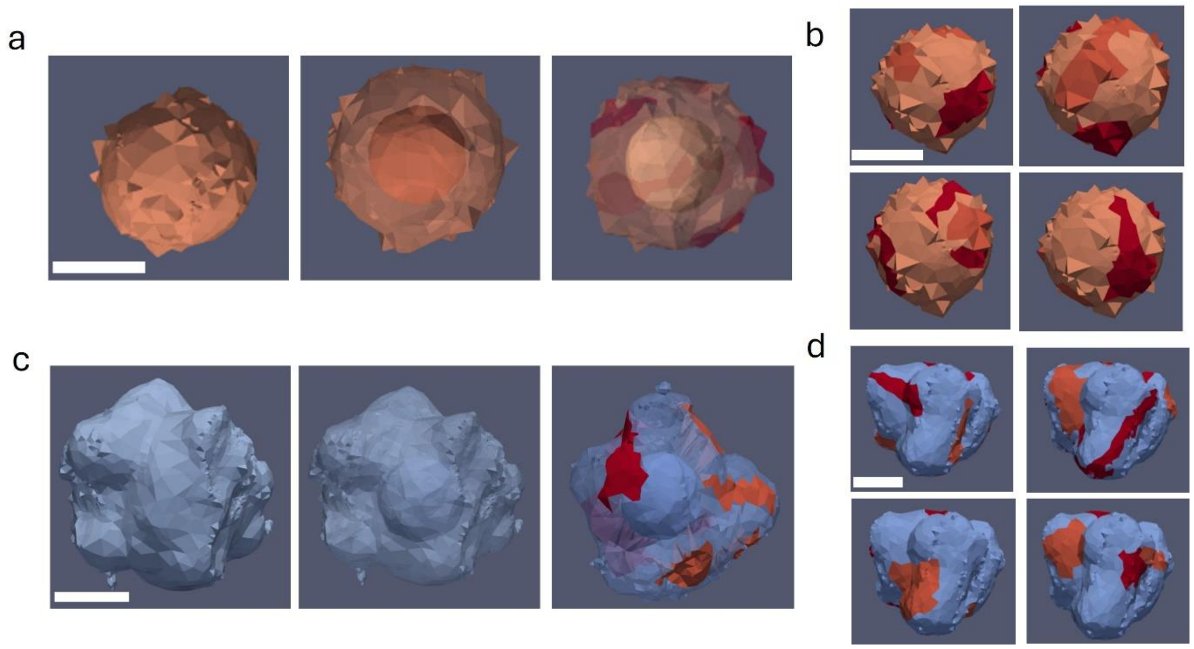

Fig. S2 (a) Three spherical models representing three scenarios: The first set presents equal homogeneity of [PS] in the tumor core and rim (left). The second set shows different but homogeneous concentrations in the core and rim (middle). The third set demonstrates different average [PS] values in the core and rim, incorporating heterogeneous concentration distributions in both (right). **(b)** Four different heterogeneity patterns for the spherical model in set 3 are labeled as model 1 (top-left), model 2 (top-right), model 3 (bottom-left), and model 4 (bottom-right). **(c)** Three non-spherical models represent the same three scenarios. The resolution for these models is the same, and the average [PS] in heterogeneous models equals the homogeneous models. **(d)** Four different heterogeneity patterns were presented for the non-spherical model in set 3, labeled as models 1 to 4 in the same order as above. White scale bars correspond to 20 mm.

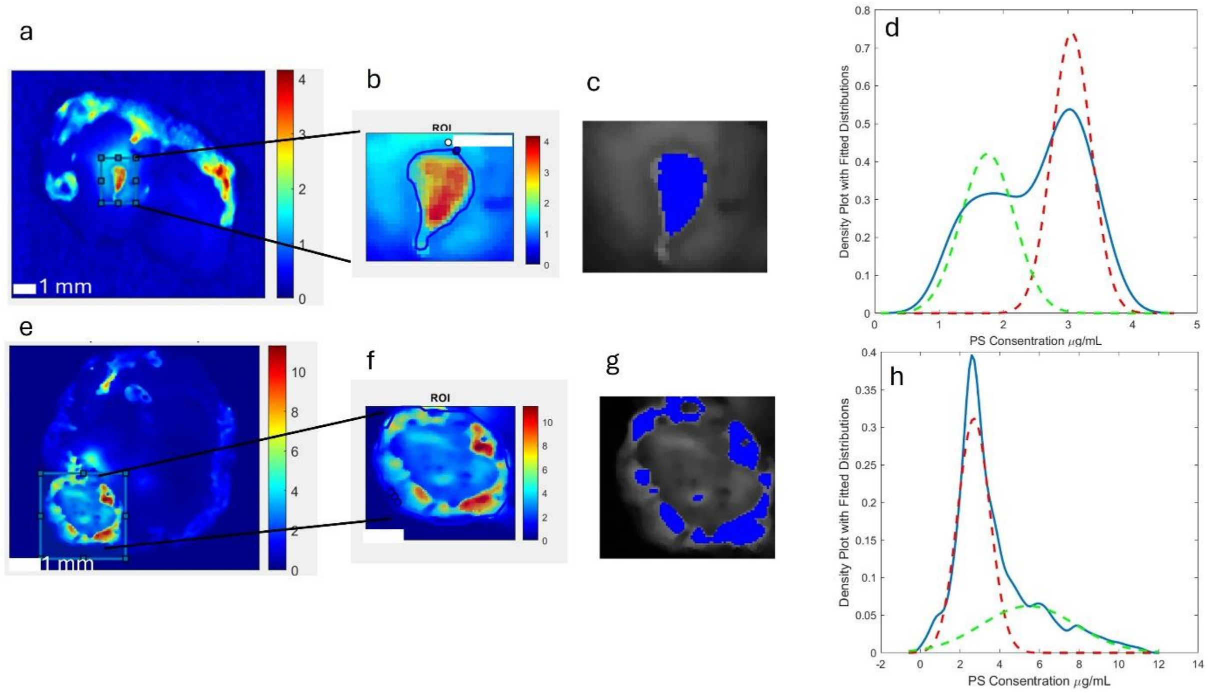

Fig. S3 Comparison of fluorescence distribution maps and density plots between small and large tumors. **(a)** Fluorescence concentration map captured by qSFDI at 0.5 mm^{-1} for a small tumor with a diameter of 1.9 mm, including selected Regions of Interest (ROI) within the tumor to reveal the concentration of Ce6 in the tissue. **(b)** A zoomed-in ROI within the small tumor. **(c)** The threshold mask identifies regions with intensity above the global threshold for the small tumor. **(d)** The density plot of pixel intensities within the small tumor shows a bimodal distribution of PS concentration with a less broad distribution. **(e)** Fluorescence concentration map for a 4.2 mm diameter tumor, with similar analysis as in (a), showing selected ROI. **(f)** Zoomed-in ROI within the large tumor. **(g)** Threshold mask identifying regions with intensity above the global threshold for the large tumor. **(h)** Density plot for the larger tumor, showing a broader distribution of PS concentration.

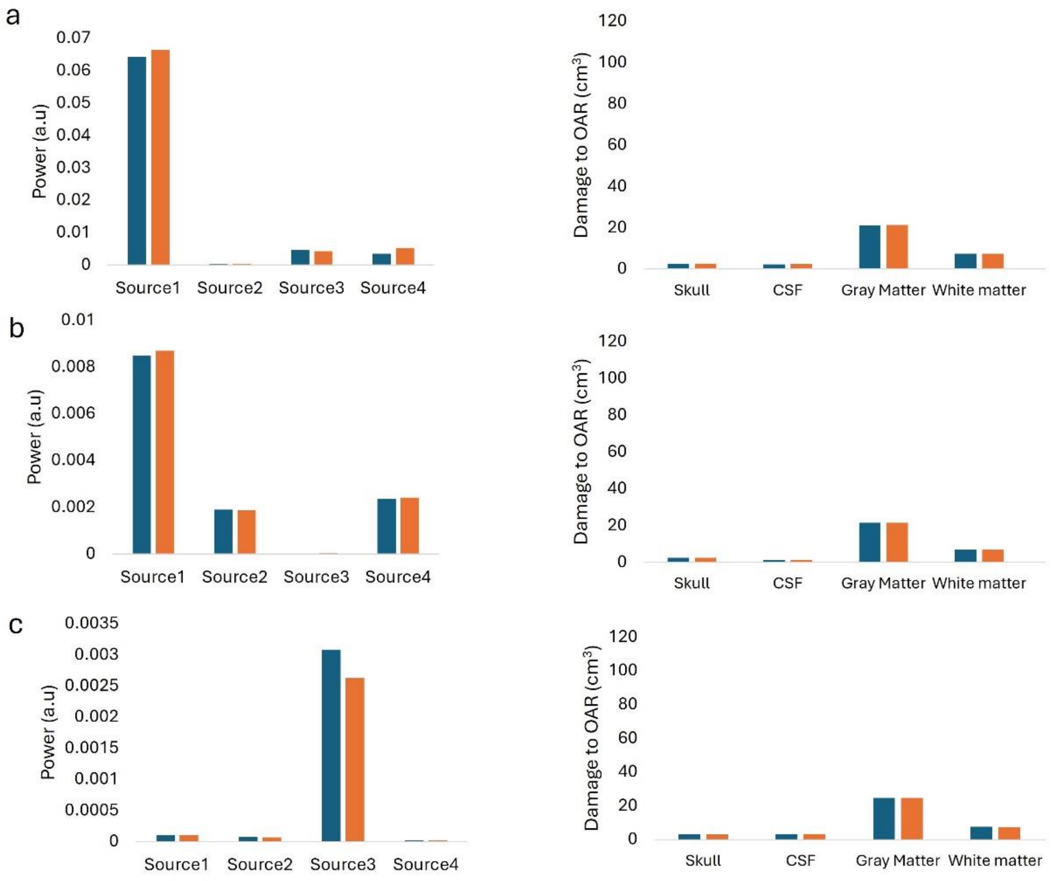

Fig. S4 PDT-SPACE results top to bottom for three high spatial resolution models. **(Left)** The source power allocations for fixed source positions with homogeneous (blue) and heterogeneous (orange) PS distributions across three different high spatial resolution models. **(Right)** The resulting potential damage to OAR (in cm^3) when 98% of the tumor is destroyed under the same conditions for the same models.

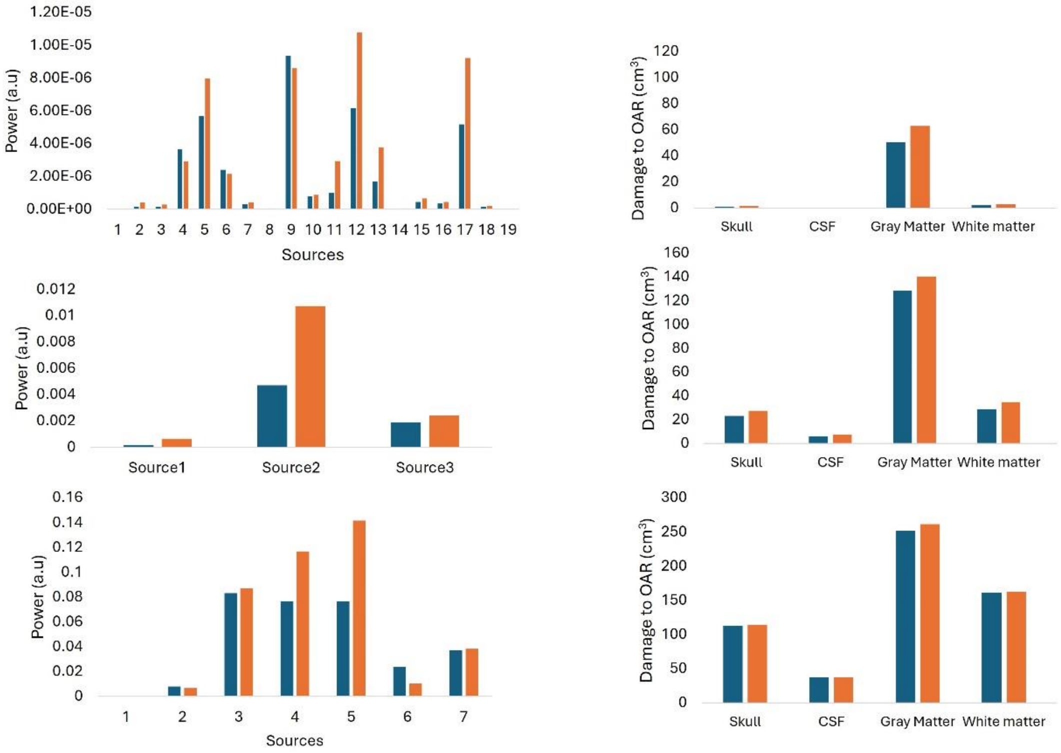

Fig. S5 PDT-SPACE results top to bottom for three low spatial resolution models. **(Left)** The source power allocations for fixed source positions with homogeneous (blue) and heterogeneous (orange) PS distributions across three different low spatial resolution models. **(Right)** The resulting potential damage to OAR (in cm³) when 98% of the tumor is destroyed under the same conditions for the same models.

Table. S1 Tumor models with corresponding tumor volumes and the number of sources used for each model. The models include low-resolution (LR), high-resolution (HR), and medium-resolution (MR) tumors with either spherical or non-spherical geometries. Tumor volumes are reported in cubic centimeters (cm^3), and the number of sources indicates the total number of light sources used for treatment planning in each tumor model.

Tumor Model	Tumor volume (cm^3)	# sources
LR Tumor 1	103.81	19
LR Tumor 2	31.77	3
LR Tumor 3	38.51	7
HR Tumor 1	23.03	4
HR Tumor 2	24.08	4
HR Tumor 3	17.55	4
MR spherical	19.41	3
MR non-Spherical	37.92	3

Table. S2 Photosensitizer distribution metrics for rats administered with different photosensitizers. The metrics include the mean and standard deviation (σ) of the first and second modes, coefficients of variation (CV) for both modes and interquartile range (IQR 10% to 90%). Group A received Porphysome as the photosensitizer, while Group B received Ce6. Rats within each group are ordered from 1 to 4.

**Metric**	**Rat 1, Group A**	**Rat 2, Group A**	**Rat 3, Group A**	**Rat 4, Group A**	**Rat 1, Group B**	**Rat 2, Group B**	**Rat 3, Group B**	**Rat 4, Group B**
Mean $_{Mode1}$	12.52	12.73	8.26	9.17	2.21	1.61	3.45	3.03
Mean $_{\sigma Mode1}$	3.36	4.60	2.62	3.35	0.76	0.48	1.06	1.25
Mean $_{Mode2}$	20.11	28.31	22.74	18.5	4.72	2.72	6.27	7.97
Mean $_{\sigma Mode2}$	3.51	8.90	5.41	4.8	1.64	0.51	1.54	3.37
CV_{Mode1}	0.26	0.36	0.31	0.36	0.34	0.30	0.30	0.41
CV_{Mode2}	0.17	0.31	0.23	0.26	0.34	0.18	0.24	0.42
IQR $_{total}$	16.20	36.12	37.06	19.79	5.88	2.46	6.31	14.38

Table. S3 Displacement comparison of source positions across different spherical models and scenarios

**Comparison**	**Source 1 (mm)**	**Source 2 (mm)**	**Source 3 (mm)**
Scenario 1 vs Scenario 2	14.75	13.05	12.7
Scenario 1 vs Scenario 3 Model 1	20.81	10.68	0.03
Scenario 1 vs Scenario 3 Model 2	23.52	19.39	0.05
Scenario 1 vs Scenario 3 Model 3	23.54	7.13	0.05
Scenario 1 vs Scenario 3 Model 4	23.53	18.24	0.03
Scenario 2 vs Scenario 3 Model 1	12.6	18.45	12.71
Scenario 2 vs Scenario 3 Model 2	14	17.29	12.72
Scenario 2 vs Scenario 3 Model 3	14.02	13.04	12.72
Scenario 2 vs Scenario 3 Model 4	14.01	26.58	12.71
